# Biaryl Phosphate‐Based Inhibitors of the Transcription Factor STAT4

**DOI:** 10.1002/cmdc.202500672

**Published:** 2025-09-09

**Authors:** Nadiya Brovchenko, Anne Maria Oelsch, Christoph Protzel, Thorsten Berg

**Affiliations:** ^1^ Institute of Organic Chemistry Leipzig University Johannisallee 29 04103 Leipzig Germany

**Keywords:** biological activity, inhibitors, protein–protein interactions, Src homology 2 domains, transcription factors

## Abstract

The transcription factor signal transducer and activator of transcription (STAT)4 is a potential target for autoimmune diseases, such as inflammatory bowel disease, multiple sclerosis, rheumatoid arthritis, and diabetes mellitus. *p*‐Biphenyl phosphate is reported as an inhibitor of the STAT4 Src homology 2 domain, and it is developed to the phosphonate‐based inhibitor Stafori‐1. Herein, structure–activity relationships of *p*‐biaryl phosphates against STAT4 and their selectivity profiles against other STAT proteins are reported. The most potent biaryl phosphate‐based inhibitor originating from this article, Stafori‐2, contains the same aryl moieties as the phosphonate Stafori‐1. However, Stafori‐2 is more potent than Stafori‐1 in fluorescence polarization assays and by isothermal titration calorimetry.

## Graphical abstract

The transcription factor signal transducer and activator of transcription (STAT)4 is a potential target for autoimmune diseases. *p*‐Biphenyl phosphate is reported as an inhibitor of the STAT4 Src homology 2 domain and it is developed to the phosphonate Stafori‐1. Here, structure–activity relationships and selectivity profiles of *p*‐biaryl phosphates are presented. The most potent *p*‐biaryl phosphate inhibitor Stafori‐2 is more potent against STAT4 than Stafori‐1.

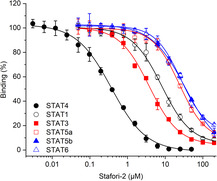

## 1 Introduction

Signal transducers and activators of transcription (STATs) are transcription factors that convey signals from the cell surface to the nucleus.^[^ ^1^ ^]^ The family member STAT4 plays key roles in autoimmune diseases, such as multiple sclerosis, rheumatoid arthritis, and type 1 diabetes.^[^ ^2^ ^]^ Upon interleukin‐12 stimulation, STAT4 is phosphorylated at tyrosine 693,^[^ ^3^ ^]^ leading to differentiation of T‐helper cells of the Th1 subgroup.^[^ ^4^ ^]^ Small‐molecule inhibitors of the STAT4 Src homology 2 (SH2) domain have the potential to serve as new therapeutic modalities for these unmet medical challenges.

**Figure 1:**
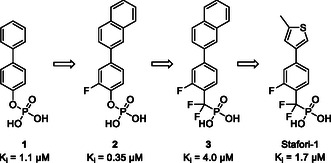
Development of the STAT4 inhibitor Stafori‐1 based on *p*‐biphenyl phosphate (**1**) as published previously.^[^ ^5^ ^]^

Inhibition of STAT4 signaling can be achieved by ligands of its protein–protein interaction domain, the SH2 domain, the key recognition element of which is phenyl phosphate as part of the side chain of a phosphorylated tyrosine residue. We discovered that *p*‐biphenyl phosphate (**1**, **Figure** **1**) is a selective inhibitor of the STAT4 SH2 domain (*K* *_i_* = 1.1 µM).^[^ ^5^ ^]^ It was further developed to the *p*‐biaryl phosphate **2** (*K* *_i_* = 0.35 µM) and its α,α‐difluorobenzyl phosphonate analog **3** (*K* *_i_* = 4.0 µM). Structural variations of the upper aromatic core of **3** led to the development of Stafori‐1, which selectively inhibits STAT4 with a *K* *_i_* of 1.7 ± 0.2 µM.^[^ ^5^ ^]^ The P2X receptor antagonists PPADS and iso‐PPADS have recently also been shown to target STAT4, along with other STAT proteins.^[^ ^6^ ^]^

## 2 Results and Discussion

The α,α‐difluorobenzyl phosphonate Stafori‐1 contains a 2‐fluoro‐substituted phenyl ring in the lower position and a 5‐methyl‐3‐thienyl group in the upper position (Figure 1). This substitution pattern was developed in the context of α,α‐difluorobenzyl phosphonates.^[^ ^5^ ^]^ In order to explore whether the optimal moieties in the lower and upper positions are influenced by the presence of the α,α‐difluorobenzyl phosphonate motif, we aimed to explore structure–activity relationships of the biaryls in the context of the phosphates.

**Scheme 1:**
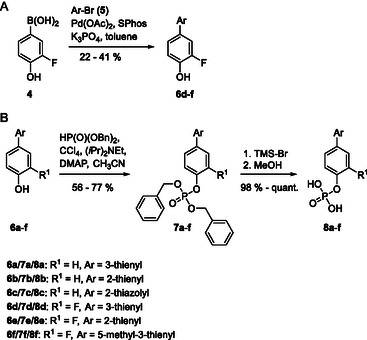
A) Synthesis of 4‐aryl phenols **6d–** **f**. Compounds **6a–** **c** were commercially available. B) Synthesis of *p*‐biaryl phosphates **8a–** **f**.

Suzuki coupling between (3‐fluoro‐4‐hydroxyphenyl)boronic acid (**4**) and aryl bromides **5** generated 4‐aryl‐2‐fluorophenols **6d–** **f** (**Scheme** **1**A), while the nonfluorinated 4‐arylphenols **6a–** **c** were commercially available. Atherton–Todd phosphorylation of *p*‐aryl phenols **6a–** **f** gave the benzyl‐protected phosphates **7a–** **f** (Scheme 1B), from which the target compounds **8a–** **f** were generated by TMS‐Br‐mediated debenzylation.

Replacing the upper phenyl ring of *p*‐biphenyl phosphate (**1**, *K* *_i_* = 1.1 ± 0.1 µM) by a 3‐thienyl moiety (**8a**) increased the activity against STAT4 as analyzed in a competitive fluorescence polarization (FP)‐based assay^[^ ^7^ ^]^ by twofold (*K* *_i_* = 0.56 ± 0.06 µM, **Table** **1** and S1, Supporting Information). Activities against STAT1, STAT3, STAT5a, and STAT6 were essentially not affected, resulting in improved overall selectivity. The derivative bearing a 2‐thienyl moiety (**8b**) was similarly active against STAT4 (*K* *_i_* = 0.50 ± 0.03 µM), but lost selectivity against STAT5a and STAT5b. The closely related 2‐thiazolyl derivative **8c** was approximately eightfold less potent against STAT4 (*K* *_i_* = 4.1 ± 0.2 µM) than **8a**. Both thienyl derivatives **8a** and **8b** were approximately as active as the previously reported 2‐naphthyl derivative **9** (*K* *_i_* = 0.44 ± 0.09 µM),^[^ ^5^ ^]^ and more potent than the 1‐naphthyl derivative **10** (*K* *_i_* = 0.84 ± 0.08 µM).^[^ ^5^ ^]^

**Table 1:** Structures of *p*‐biaryl phosphates carrying an unsubstituted phenyl ring in the lower position and their activity against STATs in FP assays. *K*_i_‐values were calculated from IC_50_‐values as described in the Supporting Information.^[^ ^12^ ^]^ Mean values ± standard deviations are given (*n* = 3).

No	Structure	STAT4 *K* *_i_* [µM]	STAT1 *K* *_i_* [µM]	STAT3 *K* *_i_* [µM]	STAT5a *K* *_i_* [µM]	STAT5b *K* *_i_* [µM]	STAT6 *K* *_i_* [µM]
**1**	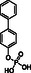	1.1 ± 0.1a)	8.1 ± 1.4a)	8.4 ± 1.0a)	22 ± 2a)	47 ± 3a)	7.3 ± 0.4a)
**8a**	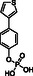	0.56 ± 0.06	9.2 ± 1.0	6.9 ± 0.3	20 ± 0.2	23 ± 1	7.8 ± 0.7
**8b**	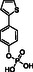	0.50 ± 0.03	8.9 ± 0.5	7.0 ± 0.5	7.3 ± 0.9	8.2 ± 0.4	9.4 ± 0.3
**8c**	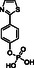	4.1 ± 0.2	38 ± 3	37 ± 1	61 ± 3	n/a	55 ± 1
**9**	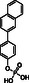	0.44 ± 0.09a)	11 ± 1a)	18 ± 1a)	27 ± 2a)	53 ± 5a)	9.3 ± 1.1a)
**10**	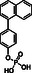	0.84 ± 0.08a)	17 ± 1	21 ± 1	4 ± 0.3	43 ± 4	9.4 ± 1.9
**11**	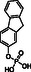	0.61 ± 0.03	6.5 ± 0.2	4.1 ± 0.4	21 ± 1	49 ± 6	12 ± 1
**12**	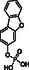	0.71 ± 0.08	16 ± 1	8.5 ± 1.2	27 ± 1	n/a	26 ± 4

a) Data taken from the literature.^[^ ^5^ ^]^ n/a: not applicable.

In the fused tricyclic phosphates **11** and **12**, the relative orientation of the two aromatic rings is fixed by a methylene group or an oxygen atom. While the methylene‐bridged compound **11** (*K* *_i_* = 0.61 ± 0.03 µM) is slightly more active than the dibenzofuran **12** (*K* *_i_* = 0.71 ± 0.08 µM) against STAT4, it has lower selectivity with regard to STAT1, STAT3, and STAT6. Overall, the thienyl derivatives **8a** and **8b** emerge as the best choice of this series, since they provide superior activities compared to *p*‐biphenyl phosphate (**1**), avoid the use of the large hydrophobic naphthyl moiety present in **9** and **10**, and allow for easier synthetic manipulation than the fused tricyclic compounds **11** and **12**.

**Figure 2:**
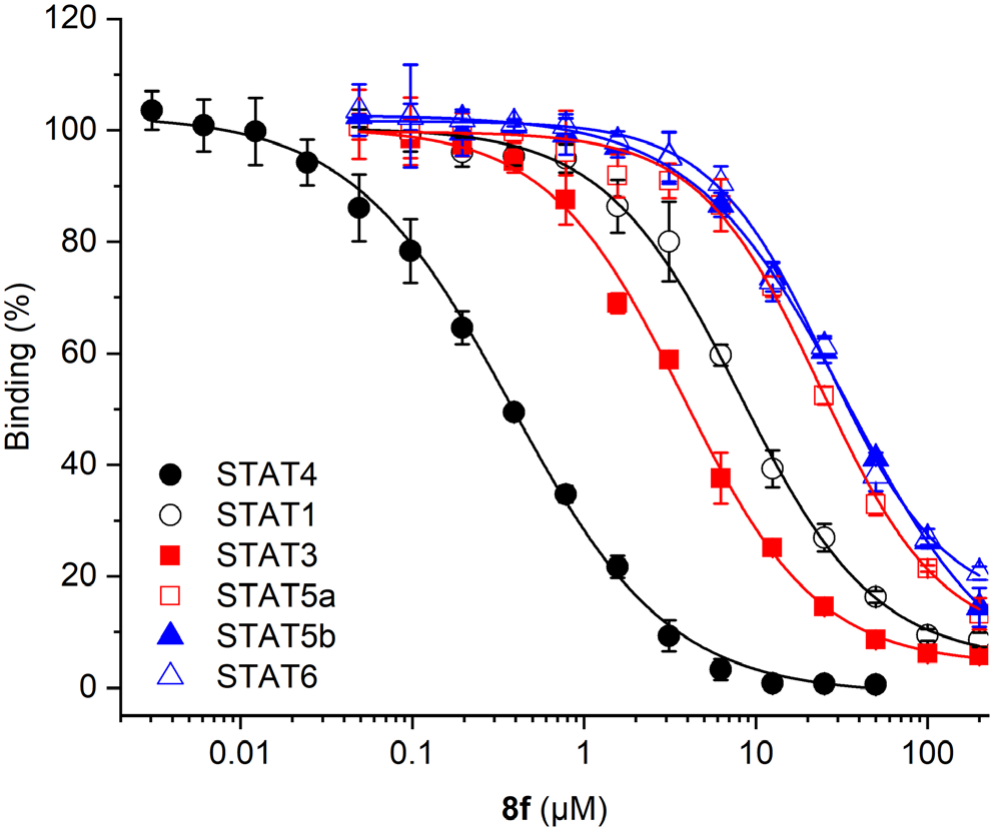
Activity of **8f** against STATs in FP assays. Error bars represent standard deviations (*n* = 3).

Introduction of a fluorine substituent in the *ortho*‐position of the lower ring of p‐biphenyl phosphate, represented by the previously reported compound **13** (*K* *_i_* = 0.56 ± 0.08 µM, **Table** **2**),^[^ ^5^ ^]^ increased the activity against STAT4 as compared to *p*‐biphenyl phosphate **1** (Table 1, *K* *_i_* = 1.1 ± 0.1 µM) by twofold. Replacing the phenyl ring in the upper position in the context of the 2‐fluorophenyl phosphate in the lower position led to the thienyl compounds **8d** (K_i_ = 0.26 ± 0.02 µM, Table 2 and S2, Supporting Information) and **8e** (*K* *_i_* = 0.30 ± 0.003 µM), both of which were more potent than **13**. Introduction of a methyl group in the 5‐position of the 3‐thienyl moiety led to compound **8f** (Table 2), the phosphate analog of the phosphonate Stafori‐1 (Figure 1). **8f** was dubbed Stafori‐2, because it turned out to be the most potent STAT4 inhibitor in FP assays (*K* *_i_* = 0.18 ± 0.03 µM, Table 2), and has good selectivity over other STAT family members (**Figure** **2**, Table S2, Supporting Information).

**Table 2:** Structures of *p*‐biaryl phosphates carrying a 2‐fluorosubstituted phenyl ring in the lower position and their activity against STATs in FP assays. *K*_i_‐values were calculated from IC_50_‐values as described in the Supporting Information.^[^ ^12^ ^]^ Mean values ± standard deviations are given (*n* = 3).

No	Structure	STAT4 *K* *_i_* [µM]	STAT1 *K* *_i_* ([µM]	STAT3 *K* *_i_* [µM]	STAT5a *K* *_i_* [µM]	STAT5b *K* *_i_* [µM]	STAT6 *K* *_i_* [µM]
**13**	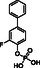	0.56 ± 0.08a)	6.3 ± 0.6a)	4.5 ± 0.3a)	28 ± 1a)	45 ± 7a)	46 ± 3a)
**8d**	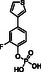	0.26 ± 0.02	n.d.	n.d.	n.d.	n.d.	n.d.
**8e**	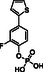	0.30 ± 0.003	n.d.	n.d.	n.d.	n.d.	n.d.
**8f** (Stafori‐2)	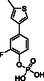	0.18 ± 0.03	4.4 ± 0.4	1.9 ± 0.1	14 ± 1	18 ± 1	17 ± 2
Stafori‐1	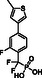	1.7 ± 0.2a)	24 ± 2a)	15 ± 2a)	39 ± 1a)	64 ± 7a)	13 ± 1a)

a) Data taken from the literature.^[^ ^5^ ^]^ n.d.: not determined.

**Figure 3:**
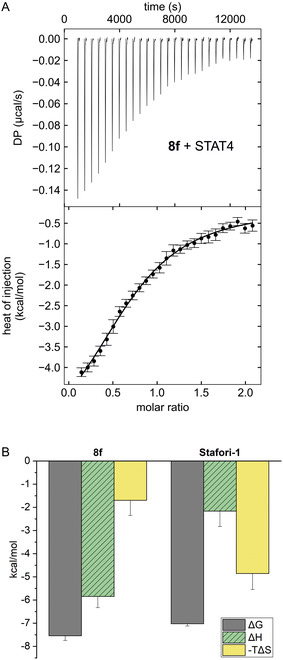
A) Thermogram of **8f** binding to STAT4 by ITC. Error bars represent integration errors assigned by the data analysis software NITPIC for the depicted individual experiment.^[^ ^11^ ^]^ B) Analysis of thermodynamic parameters for STAT4 binding of **8f** and Stafori‐1. ITC data for binding of Stafori‐1 to STAT4 were previously published.^[^ ^5^ ^]^ Mean values ± standard deviations are given (*n* = 3).

Binding of **8f** to STAT4 was validated by isothermal titration calorimetry (ITC, *K*_d_ = 3.1 ± 1.0 µM, **Figure** **3**A). No heat was generated in the control titration of **8f** into buffer (Figure S1, Supporting Information). Thus, **8f** was found to be more than twofold more potent than the corresponding phosphonate Stafori‐1 (*K*_d_ = 7.1 ± 1.1 µM).^[^ ^5^ ^]^ However, **8f** is also twofold less active in ITC against STAT4 than the naphthyl phosphate **2** (Figure 1), the binding of which to STAT4 had been investigated by ITC in the previous study (*K*_d_ = 1.3 ± 0.3 µM),^[^ ^5^ ^]^ although **8f** exhibits higher activity against STAT4 in FP assays (*K*_i_ = 0.18 ± 0.03 µM, Table 2) than **2** (*K*_i_ = 0.35 ± 0.01 µM, Figure 1).^[^ ^5^ ^]^ It is conceivable that the differences in the relative activities of **8f** and **2** are caused by the different read‐outs of the assays and the different buffer compositions. Analysis of the thermodynamic parameters of STAT4 binding derived from the ITC experiments revealed that for the phosphate **8f**, the enthalpic contribution is significantly larger than for the phosphonate Stafori‐1, while the entropic contribution toward STAT4 binding is larger for Stafori‐1 (Figure 3B). This is consistent with the notion that the bridging oxygen of the phosphate **8f** may be involved in hydrogen bond interactions with STAT4, which cannot be adequately replaced by the difluoromethylene moiety of the phosphonate Stafori‐1. Experimental validation of this hypothesis would require cocrystal structure analysis of **8f** and Stafori‐1 bound to STAT4, which has not yet been obtained.^[^ ^5^ ^]^

## 3 Conclusion

In summary, we analyzed the effect of aromatic moieties in the upper ring position of *p*‐biaryl phosphates against STAT proteins. Combining the 5‐methyl‐3‐thienyl motif in the upper position with the 2‐fluorophenyl phosphate motif in the lower position led to the most potent *p*‐biaryl phosphate **8f** (Stafori‐2), is the phosphate analog of Stafori‐1,^[^ ^5^ ^]^ which had been developed as an α,α‐difluorobenzyl phosphonate.^[^ ^8^ ^]^ Stafori‐2 is more potent against STAT4 than Stafori‐1 in FP assays and by ITC. Unlike Stafori‐1, however, Stafori‐2 is likely susceptible to hydrolysis by phosphatases, although this has yet to be investigated. Small‐molecule inhibitors of STAT4, such as Stafori‐1 or Stafori‐2, could either be used as chemical probes or further developed into molecular glues^[^ ^9^ ^]^ or proteolysis‐targeting chimeras^[^ ^10^ ^]^ against STAT proteins.

## Conflict of Interest

The authors declare no conflict of interest.

## Supporting information

Supplementary Material

## Data Availability

The data that support the findings of this study are available in the supplementary material of this article.
