# Supplementary material for: Biaryl Phosphate‐Based Inhibitors of the Transcription Factor STAT4
Source: ChemMedChem. 2025 Sep 9;20(21):e202500672. doi: 10.1002/cmdc.202500672 (PMC12597212; doi:10.1002/cmdc.202500672)
Supplement: Supplementary file 1 — Supplementary Material [file CMDC-20-e202500672-s001.pdf]

Supporting Information

Biaryl Phosphate-based Inhibitors of the Transcription Factor STAT4

Nadiya Brovchenko, Anne Maria Oelsch, Christoph Protzel and Thorsten Berg*

Table of Contents

Table S1 2

Table S2 3

Figure S1 4

Fluorescence polarization assays 4

Isothermal Titration Calorimetry 5

General synthetic methods 5

Synthesis and spectroscopic characterization of compounds 7

NMR Spectra 22

Supporting references 36

Table S1: Structures of *p*-biaryl phosphates carrying an unsubstituted phenyl ring in the lower position and their activity against STATs in FP assays.

No	Structure	STAT4	STAT1	STAT3	STAT5a	STAT5b	STAT6
**1**	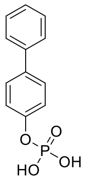	IC_{50} = 2.3 ± 0.2 µM[a]	IC_{50} = 16.4 ± 2.9 µM[a]	IC_{50} = 17.6 ± 3.0 µM[a]	IC_{50} = 42 ± 4 µM[a]	IC_{50} = 95 ± 6 µM[a]	IC_{50} = 15.0 ± 0.9 µM[a]
		K_i = 1.1 ± 0.1 µM[a]	K_i = 8.1 ± 1.4 µM[a]	K_i = 8.4 ± 1.0 µM[a]	K_i = 22 ± 2 µM[a]	K_i = 47 ± 3 µM[a]	K_i = 7.3 ± 0.4 µM[a]
**8a**	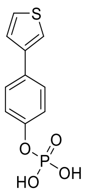	IC_{50} = 1.20 ± 0.13 µM	IC_{50} = 20.2 ± 2.3 µM	IC_{50} = 14.2 ± 0.7 µM	IC_{50} = 41 ± 0.5 µM	IC_{50} = 47 ± 3 µM	IC_{50} = 15.8 ± 1.3 µM
		K_i = 0.56 ± 0.06 µM	K_i = 9.2 ± 1.0 µM	K_i = 6.9 ± 0.3 µM	K_i = 20 ± 0.2 µM	K_i = 23 ± 1 µM	K_i = 7.8 ± 0.7 µM
**8b**	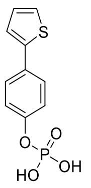	IC_{50} = 1.09 ± 0.07 µM	IC_{50} = 19.5 ± 1.2 µM	IC_{50} = 14.5 ± 0.94 µM	IC_{50} = 14.8 ± 1.81 µM	IC_{50} = 16.8 ± 0.83 µM	IC_{50} = 19.0 ± 0.67 µM
		K_i = 0.50 ± 0.03 µM	K_i = 8.9 ± 0.5 µM	K_i = 7.0 ± 0.5 µM	K_i = 7.3 ± 0.9 µM	K_i = 8.2 ± 0.4 µM	K_i = 9.4 ± 0.3 µM
**8c**	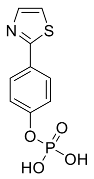	IC_{50} = 8.8 ± 0.5 µM	IC_{50} = 83 ± 6 µM	IC_{50} = 76 ± 2 µM	IC_{50} = 122 ± 7 µM	26 ± 2 % inhibition at 100 µM	IC_{50} = 112 ± 2 µM
		K_i = 4.1 ± 0.2 µM	K_i = 38 ± 3 µM	K_i = 37 ± 1 µM	K_i = 61 ± 3 µM	45 ± 1 % inhibition at 200 µM	K_i = 55 ± 1 µM
**9**	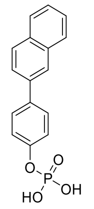	IC_{50} = 0.95 ± 0.19 µM[a]	IC_{50} = 22 ± 2 µM[a]	IC_{50} = 37 ± 2 µM[a]	IC_{50} = 55 ± 3 µM[a]	IC_{50} = 109 ±11 µM[a]	IC_{50} = 19 ± 2 µM[a]
		K_i = 0.44 ± 0.09 µM[a]	K_i = 11 ± 1 µM[a]	K_i = 18 ± 1 µM[a]	K_i = 27 ± 2 µM[a]	K_i = 53 ± 5 µM[a]	K_i = 9.3 ± 1.1 µM[a]
**10**	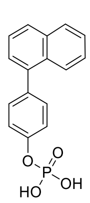	IC_{50} = 1.80 ± 0.16 µM[a]	IC_{50} = 38 ± 2 µM	IC_{50} = 43 ± 1.1 µM	IC_{50} = 8.1 ± 0.6 µM	IC_{50} = 89 ± 8 µM	IC_{50} = 19.1 ± 3.8 µM
		K_i = 0.84 ± 0.08 µM[a]	K_i = 17 ± 1 µM	K_i = 21 ± 1 µM	K_i = 4.0 ± 0.3 µM	K_i = 43 ± 4 µM	K_i = 9.4 ± 1.9 µM
**11**	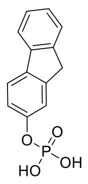	IC_{50} = 1.52 ± 0.08 µM	IC_{50} = 14.3 ± 0.4 µM	IC_{50} = 8.5 ± 0.7 µM	IC_{50} = 43 ± 2 µM	IC_{50} = 101 ± 12 µM	IC_{50} = 23 ± 2 µM
		K_i = 0.61 ± 0.03 µM	K_i = 6.5 ± 0.2 µM	K_i = 4.1 ± 0.4 µM	K_i = 21 ± 1 µM	K_i = 49 ± 6 µM	K_i = 12 ± 1 µM

No	Structure	STAT4	STAT1	STAT3	STAT5a	STAT5b	STAT6
**12**	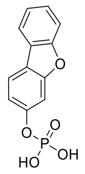	IC_{50} = 1.78 ± 0.19 µM<br><br>K_i = 0.71 ± 0.08 µM	IC_{50} = 34.3 ± 1.3 µM<br><br>K_i = 16 ± 1 µM	IC_{50} = 17.4 ± 2.5 µM<br><br>K_i = 8.5 ± 1.2 µM	IC_{50} = 55 ± 3 µM<br><br>K_i = 27 ± 1 µM	49 ± 1 % inhibition at 100 µM	IC_{50} = 53 ± 7 µM<br><br>K_i = 26 ± 4 µM

[a] Data taken from the literature. [1]

Table S2: Structures of *p*-biaryl phosphates carrying a 2-fluorosubstituted phenyl ring in the lower position and their activity against STATs in FP assays.

No	Structure	STAT4	STAT1	STAT3	STAT5a	STAT5b	STAT6
**13**	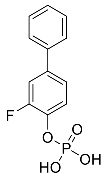	IC_{50} = 1.21 ± 0.17 µM[a]<br><br>K_i = 0.56 ± 0.08 µM[a]	IC_{50} = 12.8 ± 1.2 µM[a]<br><br>K_i = 6.3 ± 0.6 µM[a]	IC_{50} = 9.1 ± 0.6 µM[a]<br><br>K_i = 4.5 ± 0.3 µM[a]	IC_{50} = 57 ± 2 µM[a]<br><br>K_i = 28 ± 1 µM[a]	IC_{50} = 91 ± 14 µM[a]<br><br>K_i = 45 ± 7 µM[a]	IC_{50} = 90 ± 7 µM[a]<br><br>K_i = 46 ± 3 µM[a]
**8d**	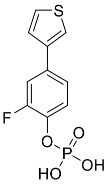	IC_{50} = 0.52 ± 0.04 µM<br><br>K_i = 0.26 ± 0.02 µM	n.d.	n.d.	n.d.	n.d.	n.d.
**8e**	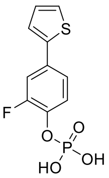	IC_{50} = 0.60 ± 0.01 µM<br><br>K_i = 0.30 ± 0.003 µM	n.d.	n.d.	n.d.	n.d.	n.d.
**8f**	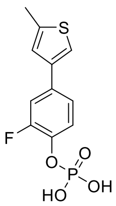	IC_{50} = 0.37 ± 0.05 µM<br><br>K_i = 0.18 ± 0.03 µM	IC_{50} = 9.0 ± 0.9 µM<br><br>K_i = 4.4 ± 0.4 µM	IC_{50} = 4.0 ± 0.2 µM<br><br>K_i = 1.9 ± 0.1 µM	IC_{50} = 28 ± 1 µM<br><br>K_i = 14 ± 1 µM	IC_{50} = 36 ± 3 µM<br><br>K_i = 18 ± 1 µM	IC_{50} = 34 ± 3 µM<br><br>K_i = 17 ± 2 µM
Stafori-1	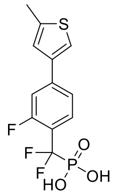	IC_{50} = 3.7 ± 0.4 µM[a]<br><br>K_i = 1.7 ± 0.2 µM[a]	IC_{50} = 48 ± 4 µM[a]<br><br>K_i = 24 ± 2 µM[a]	IC_{50} = 31 ± 4 µM[a]<br><br>K_i = 15 ± 2 µM[a]	IC_{50} = 79 ± 2 µM[a]<br><br>K_i = 39 ± 1 µM[a]	IC_{50} = 130 ± 14 µM[a]<br><br>K_i = 64 ± 7 µM[a]	IC_{50} = 26 ± 2 µM[a]<br><br>K_i = 13 ± 1 µM[a]

[a] Data taken from the literature.[1] n.d.: not determined.

Figure S1

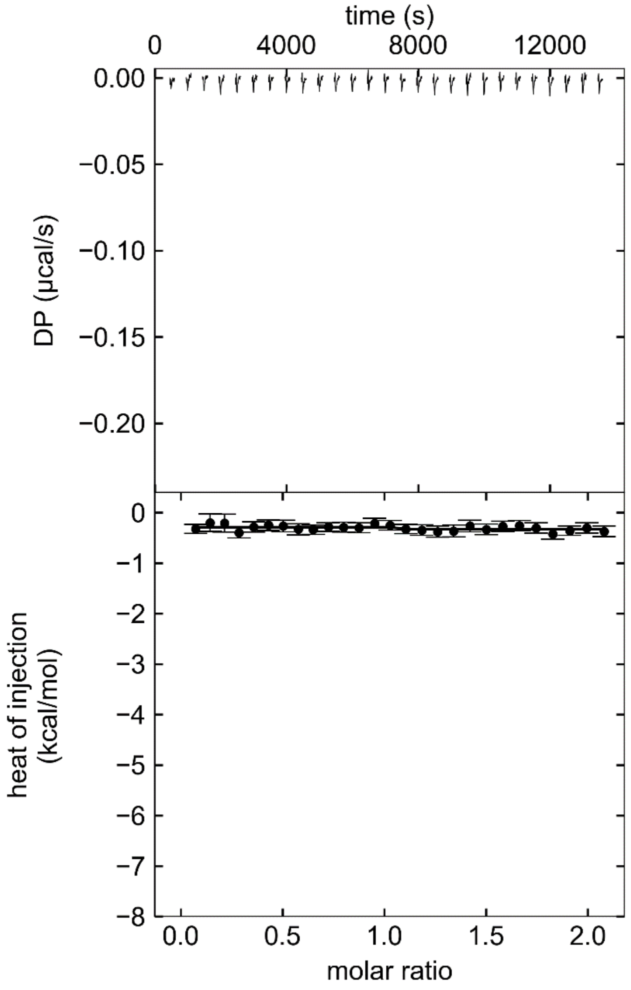

Figure S1: A) Titration of 100 µM **8f** dissolved in ITC buffer to a final DMSO concentration of 1 % (*v*/*v*) into ITC buffer supplemented with 1 % (*v*/*v*) DMSO.

Fluorescence polarization assays

Competitive fluorescence polarization assays were carried as previously described.[1] Proteins were used at the following final concentrations, which represent the approximate dissociation constant for peptide binding of the respective protein batches used in the assays: 28 nM for STAT4; between 80 nM and 92 nM for STAT1; 110 nM for STAT3; between 125 nM and 177 nM for STAT5a; between 59 nM and 85 nM for STAT5b; and 106 nM for STAT6. The following fluorescent-labeled peptides were used as tracers: 5-carboxyfluorescein-GpYLPQNID-OH for STAT4; 5-carboxyfluorescein-GpYDKPHVL for STAT1; 5-carboxyfluorescein-GpYLPQTV-NH_2 for STAT3; 5-carboxyfluorescein-GpYLVLDKW for STAT5a/b, and 5-carboxyfluorescein-GpYVPWQDLI-OH for STAT6 (all at a final concentration of 10 nM). Buffer composition: 10 mM Tris (pH 8.0), 50 mM NaCl, 1 mM EDTA, 1 mM DTT, 0.1 % Nonidet P-40 substitute, 2 % DMSO. Proteins and test compound were incubated for 1 h at room temperature before the

fluorescent-labeled peptide was added. Samples were transferred to 384-well microtiter plates and fluorescence polarization was read after an additional 60 min of incubation time. Experiments were carried out in triplicate. Percent binding was calculated based on a logarithmic curve fit using OriginPro 8G software. Conversion of IC_{50} values to K_i-values was achieved using the published equation[2] embedded in the corresponding Excel spread sheet downloaded from
https://websites.umich.edu/~shaomengwanglab/software/calc_ki/index.html.
Error bars represent standard deviations.

Isothermal Titration Calorimetry

6xHis-tagged proteins for ITC experiments were purified by affinity chromatography on His-Bind resin. Proteins were dialyzed against ITC buffer (10 mM Tris pH 8.0, 50 mM NaCl) using dialysis tubing with a 50 kDa cut-off. ITC experiments were performed using a VP-ITC Micro Calorimeter (MicroCal). **8f** was dissolved in DMSO as a 10 mM stock and was diluted 1/100 in ITC buffer (final ligand concentration: 100 µM). 1 % (*v*/*v*) DMSO was added to the buffers containing the proteins and to the reference buffer in order to prevent errors caused by the detection of dilution heat. Proteins were degassed before the experiments using a ThermoVac sample degassing station. ITC experiments were carried out using the following conditions: 10 µM STAT4, 100 µM **8f**, 28 injections with 10 µL single injection volume, 250 s initial delay, 500 s spacing between injections, 20 µcal/s reference power, 220 rpm stirring at 25 °C. A low-noise integration approach was used for data analysis with NITPIC[3-4] and SEDPHAT.[5] A one-site binding model was used for data fitting. Figures were generated using GUSSI.[6] Experiments were carried out in triplicate.

General synthetic methods

Method 1: Suzuki coupling

A *Schlenk* flask was charged with aryl bromide (1.0 eq.), the corresponding boronic acid (2.0 eq.), K_3PO_4 (3.0 eq.), $Pd(OAc)_2$ (5.0 mol%), and SPhos (L:Pd=2.5:1) under inert gas atmosphere. Dry toluene degassed by sonication was added to the mixture, which was stirred at 100° C for 20 h. The reaction was subsequently cooled, diluted with water, and extracted three times with ethyl acetate. The combined organic layers were washed with water, after which they were dried over Na_2SO_4 and filtered. The volatiles were removed under reduced pressure, and the crude product was purified by flash column chromatography.

Method 2: Benzyl phosphorylation

To prepare the desired benzyl-protected phosphate, the corresponding phenol (1.0 eq.) was dissolved in dry acetonitrile (0.125 M), followed by the addition of CCl_4 (10 eq.), DIPEA (2.0 eq.), and catalytic amounts of DMAP (0.10 eq.). Dibenzyl phosphite (1.5 eq.) was added to the mixture, which was then stirred at 0 °C for 90 min. After reaching room temperature, the reaction was stirred for a further 16–19 h. A 0.5 M KH_2PO_4 solution (3.0 mL) was introduced, and the mixture was extracted with ethyl acetate. The combined organic phases were washed with brine and water, dried over Na_2SO_4, and filtered. The solvents were removed under reduced pressure, and the crude product was purified by flash column chromatography.

Method 3: Debenzylation by TMS-Br

Dibenzyl phosphate dissolved in dry DCM (0.065 M) was cooled to 0 °C. TMS-Br (6–10 eq.) was added dropwise to the mixture, which was concurrently stirred for 2 h at the same temperature. After reaching ambient conditions, the mixture was stirred for a further 16–17 h. The reaction was then quenched with methanol, and the volatiles were removed under reduced pressure. The resulting residue was washed with methanol several times, and C_{18} reversed-phase column chromatography was used if additional purification was necessary. The product was dissolved in water and then lyophilized for isolation.

Method 4: Debenzylation by hydrogenolysis

10 wt. % Pd/C (25 mg) was added to a solution of the corresponding benzyl-protected phosphate in ethanol. The mixture was kept under an inert gas atmosphere, which was later replaced with hydrogen. After being stirred for 1 h, the reaction mixture was filtered through celite and washed with ethanol. The solvent was evaporated under reduced pressure, and C_{18} reversed-phase chromatography was used if additional purification was necessary. The product was dissolved in water and then lyophilized for isolation.

Synthesis and spectroscopic characterization of compounds

Dibenzyl 4-(thiophen-3-yl)phenyl phosphate (**7a**)

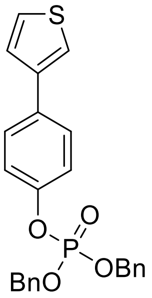

Compound **7a** was synthesized from 4-(thiophen-3-yl)phenol (**6a**, 101 mg, 0.573 mmol, 1.0 eq., purchased from ABCR) according to **Method 2**. The crude product was purified by flash column chromatography (hexane to ethyl acetate/hexane 1:4, *v*/*v*) to give **7a** as a light-yellow solid.

Yield: 181 mg (72 %).

^{1}H-NMR (400 MHz, $CDCl_3$) δ =7.53 – 7.48 (m, 2H), 7.40 – 7.37 (m, 2H), 7.37 – 7.30 (m, 11H), 7.20 – 7.14 (m, 2H), 5.14 (d, *J* = 8.4 Hz, 4H; OC*H*$_2$Ph) ppm.

^{31}P-NMR (162 MHz, $CDCl_3$) δ = -6.46 (s) ppm.

^{13}C-NMR (101 MHz, $CDCl_3$) δ = 149.8 (d, $^{2}J_{C\text{-}P}$ = 7.1 Hz; *C*OP), 141.5, 135.6 (d, $^{3}J_{C\text{-}P}$ = 7.1 Hz), 133.1 (d, $^{5}J_{C\text{-}P}$ = 1.2 Hz), 128.8, 128.7, 128.2, 127.8 (d, $^{4}J_{C\text{-}P}$ = 0.8 Hz), 126.5, 126.4, 120.5 (d, $^{3}J_{C\text{-}P}$ = 4.9 Hz), 120.4, 70.1 (d, $^{2}J_{C\text{-}P}$ = 5.8 Hz; O*C*H$_2$Ph) ppm.

HRMS (ESI) *m*/*z* $[M+H]^+$ calculated for $C_{24}H_{22}O_4PS^+$: 437.0971 , found: 437.0971.

IR (KBr): ṽ 3101 (m), 3033 (m), 2958 (w), 2909 (w), 1604 (m), 1536 (m), 1499 (s), 1455 (m) 1281 (s), 1221 (s), 1171 (s), 1055 (s), 1035 (s), 1025 (s), 1015 (s), 949 (s), 845 (s), 781 (s), 737 (s), 698 (s) cm^{-1}.

UV/Vis ($CHCl_3$): λ_{max} = 242; 263 nm.

R_f = 0.37 (ethyl acetate/hexane 3:7, *v*/*v*).

m.p.: 70 – 72 °C.

4-(Thiophen-3-yl)phenyl dihydrogen phosphate (**8a**)

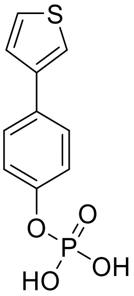

Compound **8a** was prepared from compound **7a** according to **Method 3**. Compound **7a** (51.7 mg, 0.118 mmol, 1.0 eq.) was stirred with TMS-Br (0.100 mL, 116 mg, 0.758 mmol, 6.4 eq.) in 2 mL of dry DCM for 2 h at 0 °C and then for 16 h at room temperature. The product was obtained as an off-white solid.

Yield: 31 mg (quant).

^{1}H-NMR (400 MHz, DMSO-d_6) δ =7.80 (dd, *J* = 2.9, 1.3 Hz, 1H), 7.71 – 7.66 (m, 2H), 7.63 (dd, *J* = 5.0, 2.9 Hz, 1H), 7.52 (dd, *J* = 5.0, 1.4 Hz, 1H), 7.22 – 7.17 (m, 2H) ppm.

^{31}P-NMR (162 MHz, DMSO-d_6) δ = -6.66 (s) ppm.

^{13}C-NMR (101 MHz, DMSO-d_6) δ = 150.7 (d, $^{2}J_{C-P}$ = 4.5 Hz; *C*OP), 140.8, 131.1, 127.2, 127.1, 126.2, 120.44, 120.41 ppm.

HRMS (ESI) *m*/*z* $[M-H]^-$ calculated for $C_{10}H_8O_4PS^-$: 254.9886, found: 254.9886.

IR (KBr): ṽ 3435 (br), 3098 (w), 1632 (w), 1606 (m), 1536 (m), 1502 (s), 1252 (s), 1174 (m), 1039 (s), 1012 (s), 842 (m), 779 (s) cm^{-1}.

UV/Vis (H_2O): λ_{max} = 201; 227; 263 nm.

R_f = 0.55 (acetonitrile/water 1:4, *v*/*v*).

m.p.: decomposition at 194 °C.

Dibenzyl 4-(thiophen-2-yl)phenyl phosphate (**7b**)

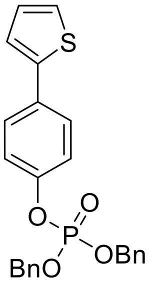

Compound **7b** was synthesized from 4-(thiophen-2-yl)phenol (**6b**, 101 mg, 0.573 mmol, 1.0 eq., purchased from ABCR) according to **Method 2**. The crude product was purified by flash column chromatography (hexane to ethyl acetate/hexane 1:4, *v*/*v*) to give **7b** as a beige solid.

Yield: 189 mg (76 %).

^{1}**H-NMR** (400 MHz, $CDCl_3$) δ = 7.54 – 7.48 (m, 2H), 7.38 – 7.30 (m, 10H), 7.27 (dd, *J* = 5.1, 1.1 Hz, 1H), 7.24 (dd, *J* = 3.6, 1.2 Hz, 1H), 7.17 – 7.12 (m, 2H), 7.07 (dd, *J* = 5.1, 3.6 Hz, 1H), 5.14 (d, *J* = 8.5 Hz, 4H; O*CH*$_2$Ph) ppm.
^{31}**P-NMR** (162 MHz, $CDCl_3$) δ = -6.16 (s) ppm.
^{13}**C-NMR** (101 MHz, $CDCl_3$) δ = 150.0 (d, $^{2}J_{C\text{-}P}$ = 7.0 Hz; *C*OP), 143.5, 135.5 (d, $^{3}J_{C\text{-}P}$ = 6.8 Hz), 131.7 (d, $^{5}J_{C\text{-}P}$ = 1.2 Hz), 128.82, 128.75, 128.2, 127.4 (d, $^{4}J_{C\text{-}P}$ = 0.7 Hz), 125.1, 123.3, 120.7 (d, $^{3}J_{C\text{-}P}$ = 4.9 Hz), 70.2 (d, $^{2}J_{C\text{-}P}$ = 5.8 Hz; O*C*H$_2$Ph) ppm.
HRMS (ESI) *m/z* $[M+Na]^+$ calculated for $C_{24}H_{21}O_4PSNa^+$: 459.0790, found: 459.0792.
IR (KBr): ṽ 3065 (w), 3036 (w), 2898 (w), 1603 (m), 1531 (m), 1498 (s), 1278 (s), 1219 (s), 1040 (s), 1013 (s), 945 (s), 846 (s), 744 (s), 698 (s) cm^{-1}.
UV/Vis ($CHCl_3$): λ_{max} = 286 nm.
$\mathbf{R_f}$ = 0.39 (ethyl acetate/hexane 3:7, *v*/*v*).
m.p.: 56 – 57 °C.

4-(Thiophen-2-yl)phenyl dihydrogen phosphate (**8b**)

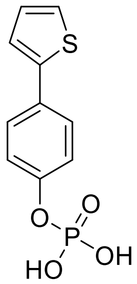

Compound **8b** was prepared from compound **7b** according to **Method 3**. Compound **7b** (51.8 mg, 0.119 mmol, 1.0 eq.) was stirred with TMS-Br (0.100 mL, 116 mg, 0.758 mmol, 6.4 eq.) in 2 mL of dry DCM for 2 h at 0 °C and then for 16 h at room temperature. The product was obtained as an off-white solid.
Yield: 31 mg (quant).
^{1}**H-NMR** (400 MHz, DMSO-d$_6$) δ = 7.65 – 7.60 (m, 2H), 7.51 (dd, *J* = 5.1, 1.2 Hz, 1H), 7.44 (dd, *J* = 3.6, 1.2 Hz, 1H), 7.23 – 7.17 (m, 2H), 7.12 (dd, *J* = 5.1, 3.6 Hz, 1H) ppm.
^{31}**P-NMR** (162 MHz, DMSO-d$_6$) δ = -6.66 (s) ppm.
^{13}**C-NMR** (101 MHz, DMSO-d$_6$) δ = 151.1 (d, $^{2}J_{C\text{-}P}$ = 4.5 Hz; *C*OP), 142.7, 129.7, 128.5, 126.6, 125.4, 123.4, 120.7 (d, $^{3}J_{C\text{-}P}$ = 3.5 Hz) ppm.
HRMS (ESI) *m/z* $[M-H]^-$ calculated for $C_{10}H_8O_4PS^-$: 254.9886, found: 254.9883.
IR (KBr): ṽ 3426 (br), 3103 (w), 3069 (w), 1605 (m), 1536 (m), 1502 (s), 1249 (s), 1179 (s), 1017 (s), 823 (s) cm^{-1}.
UV/Vis (H_2O): λ_{max} = 286 nm.
$\mathbf{R_f}$ = 0.55 (acetonitrile/water 1:4, *v*/*v*).
m.p.: decomposition at 167 °C.

Dibenzyl 4-(1,3-thiazol-2-yl)phenyl phosphate (**7c**)

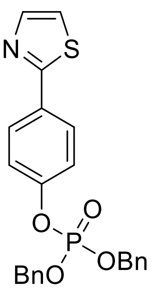

Compound **7c** was synthesized from 4-(thiazol-2-yl)phenol (**6c**, 100 mg, 0.564 mmol, 1.0 eq., purchased from ABCR) according to **Method 2**. The crude product was purified by two successive flash column chromatographies (hexane to ethyl acetate/hexane 3:7, *v*/*v*, then methanol/dichloromethane 1:39, *v*/*v*) to give **7c** as an off-white solid.

Yield: 173 mg (70 %).

^{1}H-NMR (400 MHz, $CDCl_3$) δ = 7.91 – 7.86 (m, 2H), 7.85 (d, *J* = 3.3 Hz, 1H), 7.38 – 7.30 (m, 11H), 7.22 – 7.18 (m, 2H), 5.14 (d, *J* = 8.6 Hz, 4H; OCH_2Ph) ppm.

^{31}P-NMR (162 MHz, $CDCl_3$) δ = -6.46 (s) ppm.

^{13}C-NMR (101 MHz, $CDCl_3$) δ = 167.4 (N*C*S), 151.9 (d, $^{2}J_{C\text{-}P}$ = 7.0 Hz; *C*OP), 143.9, 135.4 (d, $^{3}J_{C\text{-}P}$ = 6.7 Hz), 130.8 (d, $^{5}J_{C\text{-}P}$ = 1.2 Hz), 128.9, 128.8, 128.22, 128.15 (d, $^{4}J_{C\text{-}P}$ = 0.7 Hz), 120.7 (d, $^{3}J_{C\text{-}P}$ = 5.1 Hz), 119.0, 70.3 (d, $^{2}J_{C\text{-}P}$ = 5.8 Hz; O*C*H_2Ph) ppm.

HRMS (ESI) *m*/*z* $[M+H]^+$ calculated for $C_{23}H_{21}NO_4PS^+$: 438.0923, found: 438.0925.

IR (KBr): ṽ 3115 (w), 3080 (w), 3064 (w), 3036 (w), 2947 (w), 2895 (w), 1604 (m), 1516 (s), 1481 (s), 1275 (s), 1227 (s), 1027 (s), 1015 (s), 999 (s), 956 (s), 840 (s), 736 (s), 697 (s) cm^{-1}.

UV/Vis ($CHCl_3$): λ_{max} = 291 nm.

R_f = 0.36 (ethyl acetate/hexane 2:3, *v*/*v*).

m.p.: 61 – 65 °C.

4-(1,3-Thiazol-2-yl)phenyl dihydrogen phosphate (**8c**)

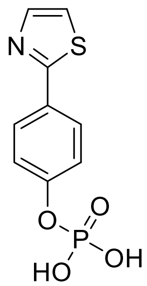

Compound **8c** was prepared from compound **7c** according to **Method 3**. Compound **7c** (50.3 mg, 0.115 mmol, 1.0 eq.) was stirred with TMS-Br (0.100 mL, 116 mg, 0.758 mmol, 6.6 eq.) in 2 mL of dry DCM for 2 h at 0 °C and then for 16 h at room temperature. The crude product was purified by C_{18} reversed-phase column chromatography (water to acetonitrile/water 1:9, *v*/*v*). Lyophilization afforded **8c** as a colorless solid.

Yield: 29 mg (98 %).

^{1}H-NMR (400 MHz, DMSO-d$_6$) δ = 7.91 – 7.85 (m, 3H), 7.72 (d, *J* = 3.3 Hz, 1H), 7.34 – 7.25 (m, 2H) ppm.

^{31}P-NMR (162 MHz, DMSO-d$_6$) δ = -6.98 (s) ppm.

^{13}C-NMR (101 MHz, DMSO-d$_6$) δ = 166.7 (N*C*S), 154.0 (d, $^2J_{C\text{-}P}$ = 4.7 Hz; *C*OP), 143.7, 128.2, 127.4, 120.6, 119.9 ppm.

HRMS (ESI) *m*/*z* [M-H]$^-$ calculated for $C_9H_7NO_4PS^-$: 255.9839, found: 255.9837.

IR (KBr): ṽ 3443 (br), 1636 (m), 1604 (m), 1515 (m), 1488 (w), 1248 (m), 1185 (m), 1098 (m), 903 (m), 753 (m) cm^{-1}.

UV/Vis (H_2O): λ_{max} = 291 nm.

R$_f$ = 0.69 (acetonitrile/water 1:4, *v*/*v*).

m.p.: 120 – 123 °C.

Dibenzyl 9*H*-fluoren-2-yl phosphate (**11a**)

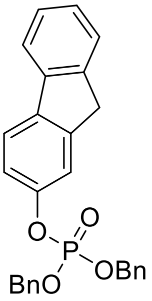

Compound **11a** was synthesized from 9*H*-fluoren-2-ol (87.0 mg, 0.477 mmol, 1.0 eq.) according to **Method 2**. The crude product was purified by flash column chromatography (hexane to ethyl acetate/hexane 1:4, *v*/*v*) to give **11a** as a light-yellow solid.

Yield: 137 mg (65 %).

^{1}H-NMR (400 MHz, $CDCl_3$) δ = 7.75 – 7.72 (m, 1H), 7.67 (d, *J* = 8.2 Hz, 1H), 7.55 – 7.51 (m, 1H), 7.40 – 7.27 (m, 13H), 7.16 (ddd, *J* = 8.2, 2.3, 1.2 Hz, 1H), 5.15 (d, *J* = 8.4 Hz, 4H; OC*H*$_2$Ph), 3.84 (s, 2H; C*H*$_2$) ppm.

^{31}P-NMR (162 MHz, $CDCl_3$) δ = -6.00 (s) ppm.

^{13}C-NMR (76 MHz, $CDCl_3$) δ = 149.8 (d, $^2J_{C\text{-}P}$ = 7.2 Hz; *C*OP), 145.0 (d, $^4J_{C\text{-}P}$ = 0.7 Hz), 143.3, 141.0, 138.9 (d, $^5J_{C\text{-}P}$ = 1.2 Hz), 135.6 (d, $^3J_{C\text{-}P}$ = 6.8 Hz), 128.8, 128.7, 128.2, 127.0, 126.7, 125.1, 120.6 (d, $^4J_{C\text{-}P}$ = 0.7 Hz), 119.8, 118.9 (d, $^3J_{C\text{-}P}$ = 5.1 Hz), 117.2 (d, $^3J_{C\text{-}P}$ = 4.6 Hz), 70.1 (d, $^2J_{C\text{-}P}$ = 5.8 Hz; O*C*H$_2$Ph), 37.1 (*C*H$_2$) ppm.

HRMS (ESI) *m*/*z* [M+H]$^+$ calculated for $C_{27}H_{24}O_4P^+$: 443.1407, found: 443.1408.

IR (KBr): ṽ 3088 (w), 3064 (w), 3034 (w), 2939 (w), 2892 (w), 1615 (w), 1585 (w), 1497 (m), 1454 (s), 1282 (s), 1053 (s), 1038 (s), 1024 (s), 960 (s), 891 (s), 770 (m), 738 (s), 695 (m) cm^{-1}.

UV/Vis ($CHCl_3$): λ_{max} = 264; 296; 305 nm.

R$_f$ = 0.18 (ethyl acetate/hexane 1:4, *v*/*v*).

m.p.: 71 – 74 °C.

9*H*-Fluoren-2-yl dihydrogen phosphate (**11**)

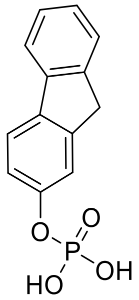

Compound **11** was prepared from compound **11a** (50.8 mg, 0.115 mmol) according to **Method 4**. The crude product was purified by C_{18} reversed-phase column chromatography (acetonitrile/water 1:4, *v*/*v*). Lyophilization afforded **11** as an off-white solid.

Yield: 19 mg (63 %).

^{1}H-NMR (400 MHz, D_2O) δ =7.90 – 7.82 (m, 2H), 7.69 – 7.64 (m, 1H), 7.50 – 7.43 (m, 2H), 7.40 – 7.34 (m, 1H), 7.29 – 7.24 (m, 1H), 3.98 (s, 2H; CH_2) ppm.

^{31}P-NMR (162 MHz, D_2O) δ = -1.14 (s) ppm.

^{13}C-NMR (101 MHz, D_2O) δ = 152.8 (d, $^{2}J_{C-P}$ = 6.4 Hz; *C*OP), 145.1, 143.6, 141.0, 136.1, 127.0, 126.4, 125.3, 120.5, 119.6, 119.3 (d, $^{3}J_{C-P}$ = 4.4 Hz), 117.5 (d, $^{3}J_{C-P}$ = 4.1 Hz), 36.6 (*C*H_2) ppm.

HRMS (ESI) *m*/*z* $[M-H]^-$ calculated for $C_{13}H_{10}O_4P^-$: 261.0322, found: 261.0323.

IR (KBr): ṽ 3435 (br), 1632 (m), 1620 (m), 1583 (w), 1487 (w), 1455 (m), 1214 (m), 1111 (m), 959 (w), 766 (w), 730 (w), 693 (w) cm^{-1}.

UV/Vis (H_2O): λ_{max} = 202; 265; 303 nm.

R_f = 0.50 (acetonitrile/water 1:4, *v*/*v*).

m.p.: decomposition at 195 °C.

Dibenzyl dibenzo[*b*,*d*]furan-3-yl phosphate (**12a**)

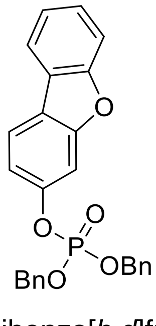

Compound **12a** was synthesized from dibenzo[*b*,*d*]furan-3-ol (76.0 mg, 0.413 mmol, 1.0 eq.) according to **Method 2**. The crude product was purified by flash column chromatography (hexane to ethyl acetate/hexane 1:4, *v*/*v*) to give **12a** as a light-yellow solid.

Yield: 83 mg (45 %).

^{1}H-NMR (300 MHz, $CDCl_3$) δ = 7.91 (ddd, *J* = 7.6, 1.4, 0.7 Hz, 1H), 7.85 – 7.80 (m, 1H), 7.56 (ddd, *J* = 8.2, 1.1, 0.7 Hz, 1H), 7.48 – 7.41 (m, 1H), 7.38 – 7.30 (m, 12H), 7.17 (ddd, *J* = 8.4, 2.1, 1.1 Hz, 1H), 5.16 (d, *J* = 8.5 Hz, 4H; OC*H*$_2$Ph) ppm.

^{31}P-NMR (162 MHz, $CDCl_3$) δ = -6.12 (s) ppm.

^{13}C-NMR (101 MHz, $CDCl_3$) δ = 156.9, 156.5 (d, $^4J_{C\text{-}P}$ = 0.7 Hz), 149.9 (d, $^2J_{C\text{-}P}$ = 7.1 Hz; *C*OP), 135.5 (d, $^3J_{C\text{-}P}$ = 6.7 Hz), 128.82, 128.75, 128.2, 127.1, 123.8, 123.1, 121.6 (d, $^5J_{C\text{-}P}$ = 1.1 Hz), 121.1 (d, $^4J_{C\text{-}P}$ = 0.7 Hz), 120.6, 115.5 (d, $^3J_{C\text{-}P}$ = 5.0 Hz), 111.8, 104.4 (d, $^3J_{C\text{-}P}$ = 5.0 Hz), 70.3 (d, $^2J_{C\text{-}P}$ = 5.8 Hz; O*C*H$_2$Ph) ppm.

HRMS (ESI) *m*/*z* $[M+H]^+$ calculated for $C_{26}H_{22}O_5P^+$: 445.1199, found: 445.1198.

IR (KBr): ṽ 3147 (m), 3035 (m), 2960 (w), 2926 (w), 2852 (w), 2808 (w), 1636 (w), 1600 (m), 1497 (m), 1457 (s), 1403 (s), 1278 (m), 1265 (m), 1037 (m), 1018 (s) 1001 (m), 971 (s), 762 (w), 747 (m), 697 (m) cm^{-1}.

UV/Vis ($CHCl_3$): λ_{max} = 244; 251; 289; 299 nm.

R$_f$ = 0.44 (ethyl acetate/hexane 3:7, *v*/*v*).

m.p.: 68 – 70 °C.

Dibenzo[*b*,*d*]furan-3-yl dihydrogen phosphate (**12**)

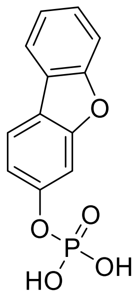

Compound **12** was prepared from compound **12a** (50.9 mg, 0.115 mmol) according to **Method 4**. The crude product was purified by C_{18} reversed-phase column chromatography (acetonitrile in water 1:4, *v*/*v*). Lyophilization afforded **12** as an off-white solid.

Yield: 27 mg (89 %).

^{1}H-NMR (400 MHz, D_2O) δ = 8.07 – 7.97 (m, 2H), 7.66 – 7.62 (m, 1H), 7.55 – 7.47 (m, 2H), 7.46 – 7.39 (m, 1H), 7.29 – 7.23 (m, 1H) ppm.

^{31}P-NMR (162 MHz, D_2O) δ = -2.57 (s) ppm.

^{13}C-NMR (75 MHz, D_2O) δ =156.3, 156.1, 152.3 (d, $^2J_{C\text{-}P}$ = 6.4 Hz; *C*OP), 126.6, 123.6, 123.1, 121.0, 120.4, 119.4, 116.1 (d, $^3J_{C\text{-}P}$ = 4.6 Hz), 111.4, 103.9 (d, $^3J_{C\text{-}P}$ = 4.3 Hz) ppm.

HRMS (ESI) *m*/*z* $[M-H]^-$ calculated for $C_{12}H_8O_5P^-$: 263.0115, found: 263.0105.

IR (KBr): ṽ 3434 (br), 1643 (m), 1603 (w), 1502 (w), 1458 (m), 1277 (m), 1191 (m), 1115 (m), 972 (w), 741 (m) cm^{-1}.

UV/Vis (H_2O): λ_{max} = 209; 250; 288; 295 nm.

R$_f$ = 0.40 (acetonitrile/water 1:4, *v*/*v*).

m.p.: decomposition at 201 °C.

2-Fluoro-4-(thiophen-3-yl)phenol (**6d**)

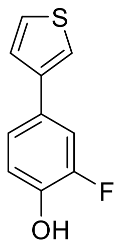

Compound **6d** was synthesized from 3-bromothiophene (200 µL, 348 mg, 2.13 mmol, 1.0 eq.), (3-fluoro-4-hydroxyphenyl)boronic acid (**4**, 669 mg, 4.29 mmol, 2.0 eq.), K_3PO_4 (1.37 g, 6.45 mmol, 3.0 eq.), $Pd(OAc)_2$ (24.2 mg, 0.108 mmol, 5.1 mol%), and SPhos (108 mg, 0.263 mmol, L:Pd=2.4:1) in 7 mL of dry and degassed toluene according to **Method 1**. The crude product was purified by flash column chromatography (ethyl acetate/hexane 1:9, *v/v*) to give **6d** as a light-yellow solid.

Yield: 139 mg (34 %).

^{1}H-NMR (400 MHz, $(CD_3)_2CO$) δ = 8.71 (s, 1H; O*H*), 7.63 (dd, *J* = 2.9, 1.4 Hz, 1H), 7.52 (dd, *J* = 5.0, 3.0 Hz, 1H), 7.48 – 7.43 (m, 2H), 7.37 (ddd, *J* = 8.4, 2.2, 1.1 Hz, 1H), 7.03 (dd, *J* = 9.2, 8.4 Hz, 1H) ppm.

^{19}F-NMR (376 MHz, $(CD_3)_2CO$) δ = -138.37 (dd, $^3J_{F\text{-}H}$ =12.2 Hz, $^4J_{F\text{-}H}$ = 9.5 Hz) ppm.

^{13}C-NMR (75 MHz, $(CD_3)_2CO$) δ = 152.5 (d, $^1J_{C\text{-}F}$ = 239.9 Hz; *C*F), 144.9 (d, $^2J_{C\text{-}F}$ = 13.1 Hz; *C*OH), 141.9 (d, $^4J_{C\text{-}F}$ = 1.9 Hz), 129.3 (d, $^3J_{C\text{-}F}$ = 6.3 Hz), 127.3, 126.8, 123.3 (d, $^3J_{C\text{-}F}$ = 3.3 Hz), 120.3, 119.0 (d, $^4J_{C\text{-}F}$ = 3.2 Hz), 114.64 (d, $^2J_{C\text{-}F}$ = 19.2 Hz) ppm.

HRMS (ESI) *m/z* $[M\text{-}H]^-$ calculated for $C_{10}H_6FOS^-$: 193.0129, found: 193.0134.

IR (KBr): ṽ 3407 (br), 3101 (w), 2926 (w), 2851 (w), 1623 (m), 1601 (m), 1543 (m), 1505 (s), 1297 (s), 1228 (m), 1210 (m), 1116 (m), 777 (s) cm^{-1}.

UV/Vis (DCM): λ_{max} = 233; 265 nm.

R_f = 0.38 (ethyl acetate/hexane 1:4, *v/v*).

m.p.: 121 – 124 °C.

Dibenzyl 2-fluoro-4-(thiophen-3-yl)phenyl phosphate (**7d**)

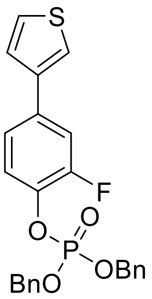

Compound **7d** was synthesized from compound **6d** (105 mg, 0.541 mmol, 1.0 eq.) according to **Method 2**. The crude product was purified by flash column chromatography (ethyl acetate/hexane 3:7, *v*/*v*) to give **7d** as a light-yellow solid.

Yield: 138 mg (56 %).

^{1}H-NMR (400 MHz, $(CD_3)_2CO$) δ = 7.81 (dd, *J* = 2.9, 1.4 Hz, 1H), 7.62 – 7.57 (m, 2H), 7.53 (dd, *J* = 5.1, 1.4 Hz, 1H), 7.52 – 7.48 (m, 1H), 7.45 – 7.33 (m, 11H), 5.23 (d, *J* = 8.3 Hz, 4H; OCH_2Ph) ppm.

^{19}F-NMR (376 MHz, $(CD_3)_2CO$) δ = -132.76 (dd, $^3J_{F-H}$ = 11.9 Hz, $^4J_{F-H}$ = 8.4 Hz) ppm.

^{31}P-NMR (162 MHz, $(CD_3)_2CO$) δ = -6.89 (d, $^4J_{P-F}$ = 1.1 Hz) ppm.

^{13}C-NMR (101 MHz, $(CD_3)_2CO$) δ = 153.6 (dd, $^1J_{C-F}$ = 247.2 Hz, $^3J_{C-P}$ = 5.9 Hz; *C*F), 140.1 (dd, $^4J_{C-F}$ = 2.1 Hz, $^6J_{C-P}$ = 0.7 Hz), 137.2 (dd, $^2J_{C-F}$ = 12.5 Hz, $^2J_{C-P}$ = 6.7 Hz; *C*OP), 135.9 (d, $^3J_{C-P}$ = 6.8 Hz), 134.3 (dd, $^3J_{C-F}$ = 7.1 Hz, $^5J_{C-P}$ = 1.6 Hz), 128.5, 128.0, 126.9, 126.1, 122.7 (dd, $^4J_{C-F}$ = 2.9 Hz, $^4J_{C-P}$ = 0.7 Hz), 122.3 (dd, $^3J_{C-F}$ = 3.5 Hz, $^3J_{C-P}$ = 1.6 Hz), 121.5, 114.5 (dd, $^2J_{C-F}$ = 19.5 Hz, $^4J_{C-P}$ = 1.1 Hz), 69.9 (d, $^2J_{C-P}$ = 5.9 Hz; O*C*H_2Ph) ppm.

HRMS (ESI) *m*/*z* $[M+H]^+$ calculated for $C_{24}H_{21}FO_4PS^+$: 455.0877, found: 455.0878.

IR (KBr): ṽ 3104 (w), 3065 (w), 3034 (w), 2958 (w), 2895 (w), 1589 (m), 1540 (m), 1500 (s), 1289 (s), 1230 (m), 1123 (m), 1038 (s), 1016 (s), 1001 (s), 953 (m), 849 (m), 740 (m), 696 (m) cm^{-1}.

UV/Vis (DCM): λ_{max} = 232; 263 nm.

R_f = 0.39 (ethyl acetate/hexane 3:7, *v*/*v*).

m.p.: 41 – 43 °C.

2-Fluoro-4-(thiophen-3-yl)phenyl dihydrogen phosphate (**8d**)

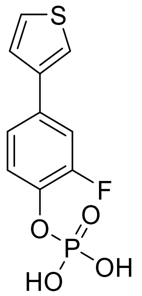

Compound **8d** was prepared from compound **7d** according to **Method 3**. Compound **7d** (31.2 mg, 0.0687 mmol, 1.0 eq.) was stirred with TMS-Br (0.0910 mL, 106 mg, 0.692 mmol, 10 eq.) in 1.4 mL of dry DCM for 2 h at 0 °C and then for 16 h at room temperature. The crude product was purified by C_{18} reversed-phase column chromatography using pure water as the eluent. Compound **8d** was obtained as a colorless solid after lyophilization.

Yield: 19 mg (quant).

^{1}H-NMR (400 MHz, D_2O+CD_3CN, *v*/*v* = 1:1) δ = 8.18 (dd, *J* = 2.9, 1.4 Hz, 1H), 8.09 – 8.06 (m, 1H), 8.06 – 7.96 (m, 4H) ppm.

^{19}F-NMR (376 MHz, D_2O+CD_3CN, *v*/*v* = 1:1) δ = -132.57 – -132.69 (m) ppm.

^{31}P-NMR (162 MHz, D_2O+CD_3CN, *v*/*v* = 1:1) δ = -3.02 (s) ppm.

^{13}C-NMR (75 MHz, D_2O+CD_3CN, *v*/*v* = 1:1) δ = 154.8 (dd, $^1J_{C\text{-}F}$ = 244.3 Hz, $^3J_{C\text{-}P}$ = 6.1 Hz; *C*F), 141.51 – 141.45 (m), 141.0 (dd, $^2J_{C\text{-}F}$ = 11.9 Hz, $^2J_{C\text{-}P}$ = 6.2 Hz; *C*OP), 132.2 (dd, $^3J_{C\text{-}F}$ = 7.0 Hz, $^5J_{C\text{-}P}$ = 1.1 Hz), 127.9, 127.0, 123.7 (dd, $^4J_{C\text{-}F}$ = 2.8 Hz, $^4J_{C\text{-}P}$ = 1.9 Hz), 123.0 (dd, $^3J_{C\text{-}F}$ = 3.5 Hz, $^3J_{C\text{-}P}$ = 1.1 Hz), 121.5, 114.8 (dd, $^2J_{C\text{-}F}$ = 20.1 Hz, $^4J_{C\text{-}P}$ = 0.8 Hz) ppm.

HRMS (ESI) *m*/*z* $[M\text{-}H]^-$ calculated for $C_{10}H_7FO_4PS^-$: 272.9792, found: 272.9793.

IR (KBr): ṽ 3135 (br), 3034 (s), 1626 (m), 1584 (m), 1542 (m), 1508 (s), 1201 (s), 1287 (m), 1235 (m), 1123 (m), 1041 (m), 845 (m), 777 (s) cm^{-1}.

UV/Vis (H_2O): λ_{max} = 201; 227; 262 nm.

R_f = 0.43 (acetonitrile/water 1:4, *v*/*v*).

m.p.: decomposition at 189 °C.

<u>2-Fluoro-4-(thiophen-2-yl)phenol (**6e**)</u>

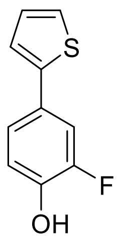

Compound **6e** was synthesized from 2-bromothiophene (200 µL, 348 mg, 2.13 mmol, 1.0 eq.), (3-fluoro-4-hydroxyphenyl)boronic acid (**4**, 669 mg, 4.29 mmol, 2.0 eq.), K_3PO_4 (1.37 g, 6.45 mmol, 3.0 eq.), $Pd(OAc)_2$ (24.0 mg, 0.107 mmol, 5.0 mol%), and SPhos (110 mg, 0.268 mmol, L:Pd=2.5:1) in 7 mL of dry and degassed toluene according to **Method 1**. The crude product was purified by flash column chromatography (ethyl acetate/hexane 1:9, *v*/*v*) to give **6e** as a light-yellow solid.

Yield: 168 mg (41 %).

^{1}H-NMR (300 MHz, $(CD_3)_2CO$) δ = 8.86 (s, 1H; O*H*), 7.44 – 7.29 (m, 4H), 7.10 – 7.00 (m, 2H) ppm.

^{19}F-NMR (282 MHz, $(CD_3)_2CO$) δ = -137.95 (dd, $^3J_{F\text{-}H}$ = 12.0 Hz, $^4J_{F\text{-}H}$ = 9.4 Hz) ppm.

^{13}C-NMR (75 MHz, $(CD_3)_2CO$) δ = 152.5 (d, $^1J_{C\text{-}F}$ = 240.5 Hz; *C*F), 145.4 (d, $^2J_{C\text{-}F}$ = 12.9 Hz; *C*OH), 143.9 (d, $^4J_{C\text{-}F}$ = 2.1 Hz), 129.0, 127.8 (d, $^3J_{C\text{-}F}$ = 6.8 Hz), 125.2, 123.6, 122.9 (d, $^3J_{C\text{-}F}$ = 3.2 Hz), 119.2 (d, $^4J_{C\text{-}F}$ = 3.2 Hz), 114.1 (d, $^2J_{C\text{-}F}$ = 19.8 Hz) ppm.

HRMS (ESI) *m*/*z* $[M\text{-}H]^-$ calculated for $C_{10}H_6FOS^-$: 193.0129, found: 193.0131.

IR (KBr): ṽ 3407 (br), 3102 (w), 3069 (w), 1628 (m), 1598 (m), 1538 (s), 1507 (s), 1299 (s), 1227 (m), 1112 (m), 814 (s), 691 (s) cm^{-1}.

UV/Vis (DCM): λ_{max} = 230; 289 nm.

R_f = 0.24 (ethyl acetate/hexane 1:9, *v*/*v*).

m.p.: 92 – 95 °C.

Dibenzyl 2-fluoro-4-(thiophen-2-yl)phenyl phosphate (**7e**)

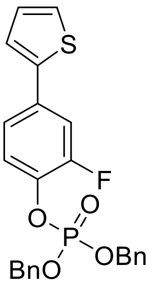

Compound **7e** was synthesized from compound **6e** (105 mg, 0.541 mmol, 1.0 eq.) according to **Method 2**. The crude product was purified by flash column chromatography (ethyl acetate/hexane 3:7, *v*/*v*) to give **7e** as a light-yellow solid.

Yield: 144 mg (59 %).

^{1}H-NMR (400 MHz, $(CD_3)_2CO$) δ = 7.57 – 7.53 (m, 1H), 7.52 – 7.49 (m, 2H), 7.46 – 7.32 (m, 12H), 7.14 (dd, J = 5.1, 3.7 Hz, 1H), 5.23 (d, J = 8.4 Hz, 4H; OCH_2Ph) ppm.

^{19}F-NMR (377 MHz, $(CD_3)_2CO$) δ = -132.33 (ddd, $^3J_{F\text{-}H}$ = 11.5 Hz, $^4J_{F\text{-}H}$ = 7.3 Hz, $^4J_{F\text{-}P}$ = 1.1 Hz) ppm.

^{31}P-NMR (162 MHz, $(CD_3)_2CO$) δ = -6.93 (d, $^4J_{P\text{-}F}$ = 1.1 Hz) ppm.

^{13}C-NMR (101 MHz, $(CD_3)_2CO$) δ = 153.6 (dd, $^1J_{C\text{-}F}$ = 247.9 Hz, $^3J_{C\text{-}P}$ = 6.0 Hz; *C*F), 141.7 (dd, $^4J_{C\text{-}F}$ = 2.4 Hz, $^6J_{C\text{-}P}$ = 0.9 Hz), 137.5 (dd, $^2J_{C\text{-}F}$ = 12.5 Hz, $^2J_{C\text{-}P}$ = 6.7 Hz; *C*OP), 135.9 (d, $^3J_{C\text{-}P}$ = 6.8 Hz), 132.9 (dd, $^3J_{C\text{-}F}$ = 7.3 Hz, $^5J_{C\text{-}P}$ = 1.7 Hz), 128.54, 128.52, 128.4, 128.1, 126.0, 124.5, 123.0 (dd, $^4J_{C\text{-}F}$ = 3.0 Hz, $^4J_{C\text{-}P}$ = 0.9 Hz), 121.8 (dd, $^3J_{C\text{-}F}$ = 3.5 Hz, $^3J_{C\text{-}P}$ = 1.6 Hz), 113.8 (dd, $^2J_{C\text{-}F}$ = 20.3 Hz, $^4J_{C\text{-}P}$ = 1.1 Hz), 69.9 (d, $^2J_{C\text{-}P}$ = 5.9 Hz; O*C*H_2Ph) ppm.

HRMS (ESI) *m*/*z* $[M+H]^+$ calculated for $C_{24}H_{21}FO_4PS^+$: 455.0877, found: 455.0877.

IR (KBr): ṽ 3091 (w), 3068 (w), 3035 (w), 2955 (w), 2896 (w), 1589 (m), 1536 (m), 1505 (s), 1271 (s), 1235 (s), 1119 (m), 1058 (s), 1035 (s), 1024 (s), 999 (s), 935 (s), 870 (s), 737 (s), 697 (s) cm^{-1}.

UV/Vis (MeOH): λ_{max} = 208; 287 nm.

R_f = 0.40 (ethyl acetate/hexane 3:7, *v*/*v*).

m.p.: 59 – 61 °C.

2-Fluoro-4-(thiophen-2-yl)phenyl dihydrogen phosphate (**8e**)

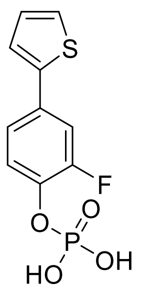

Compound **8e** was prepared from compound **7e** according to **Method 3**. Compound **7e** (30.1 mg, 0.0662 mmol, 1.0 eq.) was stirred with TMS-Br (0.0890 mL, 103 mg, 0.673 mmol, 10 eq.) in 1.3 mL of dry DCM for 2 h at 0 °C and then for 16 h at room temperature. The crude product was purified by C_{18} reversed-phase column chromatography using pure water as the eluent. Compound **8e** was obtained as a colorless solid after lyophilization.

Yield: 18 mg (quant.)

^{1}H-NMR (400 MHz, D_2O+CD_3CN, *v*/*v* = 1:1) δ = 8.01 – 7.96 (m, 1H), 7.94 – 7.91 (m, 3H), 7.90 (dd, *J* = 3.6, 1.2 Hz, 1H), 7.64 (dd, *J* = 5.1, 3.6 Hz, 1H) ppm.

^{19}F-NMR (376 MHz, D_2O+CD_3CN, *v*/*v* = 1:1) δ = -131.98 – -132.08 (m) ppm.

^{31}P-NMR (162 MHz, D_2O+CD_3CN, *v*/*v* = 1:1) δ = -4.60 (s) ppm.

^{13}C-NMR (101 MHz, D_2O+CD_3CN, *v*/*v* = 1:1) δ =153.6 (dd, $^{1}J_{C\text{-}F}$ = 245.6 Hz, $^{3}J_{C\text{-}P}$ = 6.0 Hz; *C*F), 142.1 (dd, $^{4}J_{C\text{-}F}$ = 2.4 Hz, $^{6}J_{C\text{-}P}$ = 0.8 Hz), 139.6 (dd, $^{2}J_{C\text{-}F}$ = 12.0 Hz, $^{2}J_{C\text{-}P}$ = 6.4 Hz; *C*OP), 130.2 (dd, $^{3}J_{C\text{-}F}$ = 7.3 Hz, $^{5}J_{C\text{-}P}$ = 1.3 Hz), 128.2, 125.3, 123.6, 122.8 (dd, $^{4}J_{C\text{-}F}$ = 3.0 Hz, $^{4}J_{C\text{-}P}$ = 1.6 Hz), 121.4 (dd, $^{3}J_{C\text{-}F}$ = 3.4 Hz, $^{3}J_{C\text{-}P}$ = 1.4 Hz), 113.2 (dd, $^{2}J_{C\text{-}F}$ = 20.6 Hz, $^{4}J_{C\text{-}P}$ = 0.8 Hz) ppm.

HRMS (ESI) *m*/*z* $[M-H]^-$ calculated for $C_{10}H_7FO_4PS^-$: 272.9792, found: 272.9792.

IR (KBr): ṽ 3127 (br), 3036 (m), 1627 (m), 1584 (w), 1539 (w), 1508 (m), 1400 (m), 1295 (m), 1237 (w), 1120 (w), 1044 (w), 924 (m) cm^{-1}.

UV/Vis (H_2O): λ_{max} = 201; 287 nm.

R_f = 0.43 (acetonitrile/water 1:4, *v*/*v*).

m.p.: decomposition at 189 °C.

2-Fluoro-4-(5-methylthiophen-3-yl)phenol (**6f**)

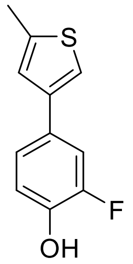

Compound **6f** was synthesized from 4-bromo-2-methylthiophene (220 µL, 348 mg, 1.97 mmol, 1.0 eq.), (3-fluoro-4-hydroxyphenyl)boronic acid (**4**, 612 mg, 3.93 mmol, 2.0 eq.), K_3PO_4 (1.25 g, 5.89 mmol, 3.0 eq.), $Pd(OAc)_2$ (23.0 mg, 0.102 mmol, 5.2 mol%), and SPhos (105 mg, 0.256 mmol, L:Pd=2.5:1) in 6.6 mL of dry and degassed toluene according to **Method 1**. The crude product was purified by flash column chromatography (hexane to ethyl acetate/hexane 1:4, *v/v*) to give **6f** as a light-yellow solid.

Yield: 90 mg (22 %).

^{1}H-NMR (400 MHz, $(CD_3)_2CO$) δ = 8.69 (s, 1H; O*H*), 7.39 (dd, $^3J_{H\text{-}F}$ = 12.5 Hz, $^4J_{H\text{-}H}$ = 2.2 Hz, 1H), 7.34 (d, *J* = 1.5 Hz, 1H), 7.31 (ddd, *J* = 8.3, 2.2, 1.0 Hz, 1H), 7.13 (h, *J* = 1.2 Hz, 1H), 7.00 (dd, $^4J_{H\text{-}F}$ = 9.2 Hz, $^3J_{H\text{-}H}$ = 8.4 Hz, 1H), 2.49 (d, *J* = 1.1 Hz, 3H; C*H*$_3$) ppm.

^{19}F-NMR (376 MHz, $(CD_3)_2CO$) δ = -139.40 (dd, $^3J_{F\text{-}H}$ = 12.5 Hz, $^4J_{F\text{-}H}$ = 9.3 Hz) ppm.

^{13}C-NMR (101 MHz, $(CD_3)_2CO$) δ = 152.5 (d, $^1J_{C\text{-}F}$ = 239.7 Hz; *C*F), 144.8 (d, $^2J_{C\text{-}F}$ = 13.1 Hz; *C*OH), 141.6 (d, $^4J_{C\text{-}F}$ = 1.9 Hz), 141.2 (*C*CH$_3$), 129.6 (d, $^3J_{C\text{-}F}$ = 6.5 Hz), 125.3, 123.0 (d, $^4J_{C\text{-}F}$ = 3.1 Hz), 118.9 (d, $^3J_{C\text{-}F}$ = 3.2 Hz), 118.1, 114.4 (d, $^2J_{C\text{-}F}$ = 19.5 Hz), 15.3 (C*C*H$_3$) ppm.

HRMS (ESI) *m*/*z* $[M\text{-}H]^-$ calculated for $C_{11}H_8FOS^-$: 207.0285, found: 207.0281.

IR (KBr): ṽ 3434 (br), 3105 (w), 2919 (w), 1625 (m), 1601 (m), 1556 (m), 1520 (s), 1295 (m), 1208 (m), 742 (m) cm^{-1}.

UV/Vis (MeOH): λ_{max} = 205; 235; 269 nm.

R$_f$ = 0.41 (ethyl acetate/hexane 1:4, *v*/*v*).

m.p.: 169 °C.

<u>Dibenzyl 2-fluoro-4-(5-methylthiophen-3-yl)phenyl phosphate (**7f**)</u>

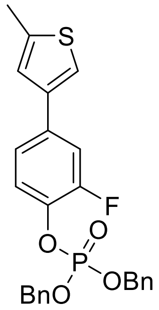

Compound **7f** was synthesized from compound **6f** (68.9 mg, 0.331 mmol, 1.0 eq.) according to **Method 2**. The crude product was purified by flash column chromatography (hexane to ethyl acetate/hexane 1:4, *v*/*v*) to give **7f** as a colorless solid.

Yield: 120 mg (77 %).

^{1}H-NMR (400 MHz, $(CD_3)_2CO$) δ = 7.57 – 7.51 (m, 2H), 7.48 – 7.30 (m, 12H), 7.22 – 7.19 (m, 1H), 5.22 (d, *J* = 8.4 Hz, 4H; OC*H*$_2$Ph), 2.51 (d, *J* = 1.1 Hz, 3H; C*H*$_3$) ppm.

^{19}F-NMR (377 MHz, $(CD_3)_2CO$) δ = -132.87 (dd, $^3J_{F\text{-}H}$ = 12.1 Hz, $^4J_{F\text{-}H}$ = 8.1 Hz) ppm.

^{31}P-NMR (162 MHz, $(CD_3)_2CO$) δ = -6.89 (d, $^4J_{P\text{-}F}$ = 1.1 Hz) ppm.

^{13}C-NMR (101 MHz, $(CD_3)_2CO$) δ = 154.5 (dd, $^{1}J_{C\text{-}F}$ = 247.1 Hz, $^{3}J_{C\text{-}P}$ = 6.0 Hz; *C*F), 141.7, 140.6 (dd, $^{4}J_{C\text{-}F}$ = 2.1 Hz, $^{6}J_{C\text{-}P}$ = 0.7 Hz), 138.0 (dd, $^{2}J_{C\text{-}F}$ = 12.6 Hz, $^{2}J_{C\text{-}P}$ = 6.7 Hz; *C*OP), 136.9 (d, $^{3}J_{C\text{-}P}$ = 6.9 Hz), 135.5 (dd, $^{3}J_{C\text{-}F}$ = 7.0 Hz, $^{5}J_{C\text{-}P}$ = 1.6 Hz), 129.4, 128.9, 125.3, 123.6 (dd, $^{4}J_{C\text{-}F}$ = 2.9 Hz, $^{4}J_{C\text{-}P}$ = 0.8 Hz), 123.0 (dd, $^{3}J_{C\text{-}F}$ = 3.5 Hz, $^{3}J_{C\text{-}P}$ = 1.6 Hz), 120.2, 115.1 (dd, $^{2}J_{C\text{-}F}$ = 19.5 Hz, $^{4}J_{C\text{-}P}$ = 1.1 Hz), 70.8 (d, $^{2}J_{C\text{-}P}$ = 5.6 Hz; O*C*H_2Ph), 15.3 (*C*H_3) ppm.

HRMS (ESI) *m/z* $[M+H]^+$ calculated for $C_{25}H_{23}FO_4PS^+$: 469.1033, found: 469.1033.

IR (KBr): ṽ 3102 (w), 2955 (w), 2927 (w), 2895 (w), 1589 (m), 1552 (m), 1513 (s), 1277 (s), 1228 (s), 1012 (s), 999 (s), 925 (s), 741 (s), 700 (m) cm^{-1}.

UV/Vis (MeOH): λ_{max} = 209; 233; 264 nm.

R_f = 0.21 (ethyl acetate/hexane 1:4, *v*/*v*).

m.p.: 86 °C.

2-Fluoro-4-(5-methylthiophen-3-yl)phenyl dihydrogen phosphate (**8f**)

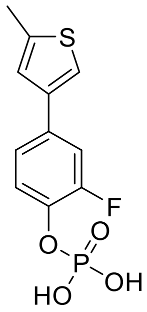

Compound **8f** was prepared from compound **7f** according to **Method 3**. Compound **7f** (30.0 mg, 0.064 mmol, 1.0 eq.) was stirred with TMS-Br (0.0850 mL, 98.6 mg, 0.644 mmol, 10 eq.) in 1.3 mL of dry DCM for 2 h at 0 °C and then for 17 h at room temperature. The crude product was purified by C_{18} reversed-phase column chromatography (water to acetonitrile/water 1/4, *v*/*v*) to afford **8f** as a colorless solid after lyophilization.

Yield: 18 mg (98 %).

^{1}H-NMR (400 MHz, D_2O) δ = 7.52 – 7.46 (m, 1H), 7.45 – 7.38 (m, 3H), 7.22 – 7.20 (m, 1H), 2.53 (d, *J* = 1.1 Hz, 3H; C*H*$_3$) ppm.

^{19}F-NMR (377 MHz, D_2O) δ = -129.15 – -129.25 (m) ppm.

^{31}P-NMR (162 MHz, D_2O) δ = -1.35 (s) ppm.

^{13}C-NMR (75 MHz, D_2O) δ = 154.0 (dd, $^{1}J_{C\text{-}F}$ = 244.1 Hz, $^{3}J_{C\text{-}P}$ = 5.6 Hz; *C*F), 142.0, 140.0 (d, $^{4}J_{C\text{-}F}$ = 1.8 Hz), 139.5 (dd, $^{2}J_{C\text{-}F}$ = 11.9 Hz, $^{2}J_{C\text{-}P}$ = 6.3 Hz; *C*OP), 131.8 – 131.7 (m), 124.0, 122.8 (dd, $^{4}J_{C\text{-}F}$ = 2.8 Hz, $^{4}J_{C\text{-}P}$ = 1.7 Hz), 121.8 – 121.7 (m), 118.5, 114.0 – 113.7 (m), 14.3 (*C*H_3) ppm.

HRMS (ESI) *m/z* $[M-H]^-$ calculated for $C_{11}H_9FO_4PS^-$: 286.9949, found: 286.9951.

IR (KBr): ṽ 3125 (br), 3011 (w), 2920 (w), 2852 (w), 1628 (m), 1587 (w), 1557 (w), 1525 (m), 1400 (m), 1233 (m), 1120 (m), 913 (m), 743 (m) cm^{-1}.

UV/Vis (H_2O): λ_{max} = 200; 233; 265 nm.

R_f = 0.30 (acetonitrile/water 1:4, *v*/*v*).

m.p.: decomposition at 187 °C.

NMR Spectra

^{1}H NMR of compound **8a**

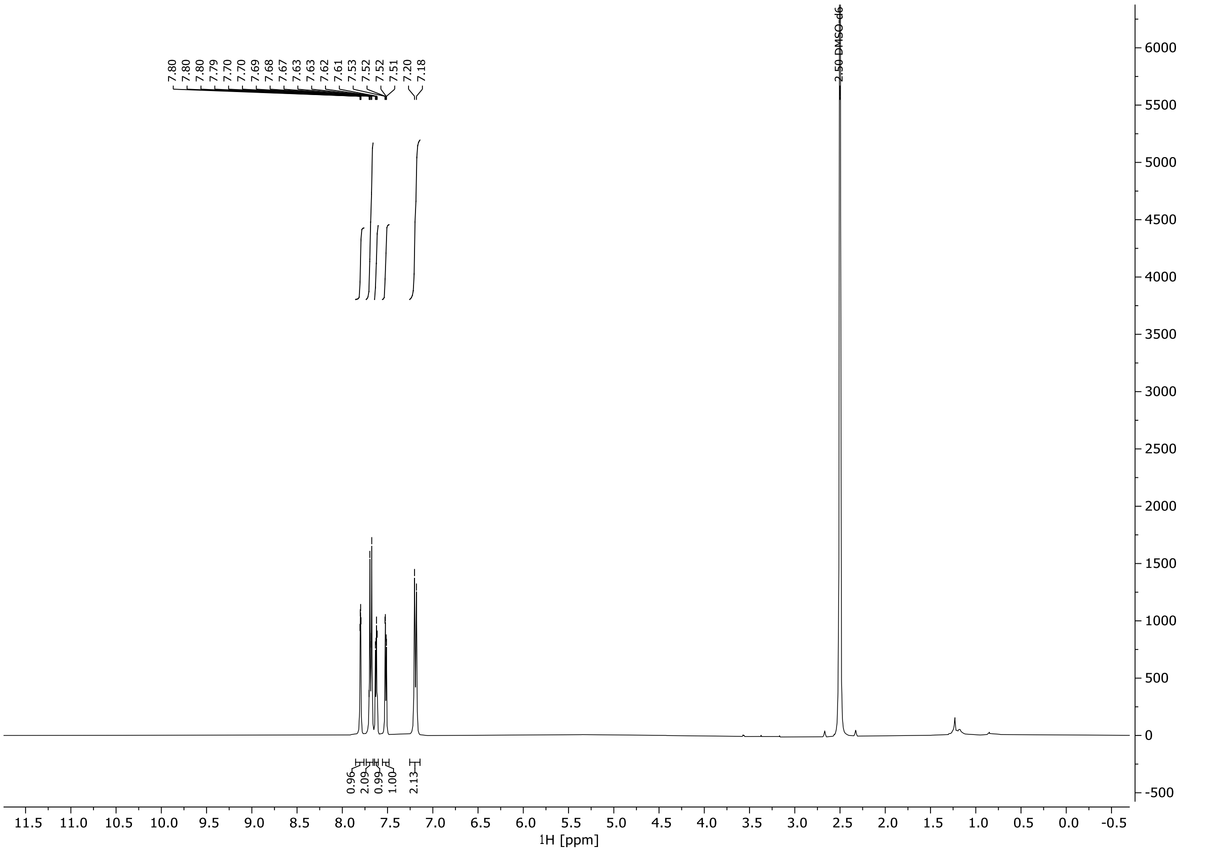

^{13}P NMR of compound **8a**

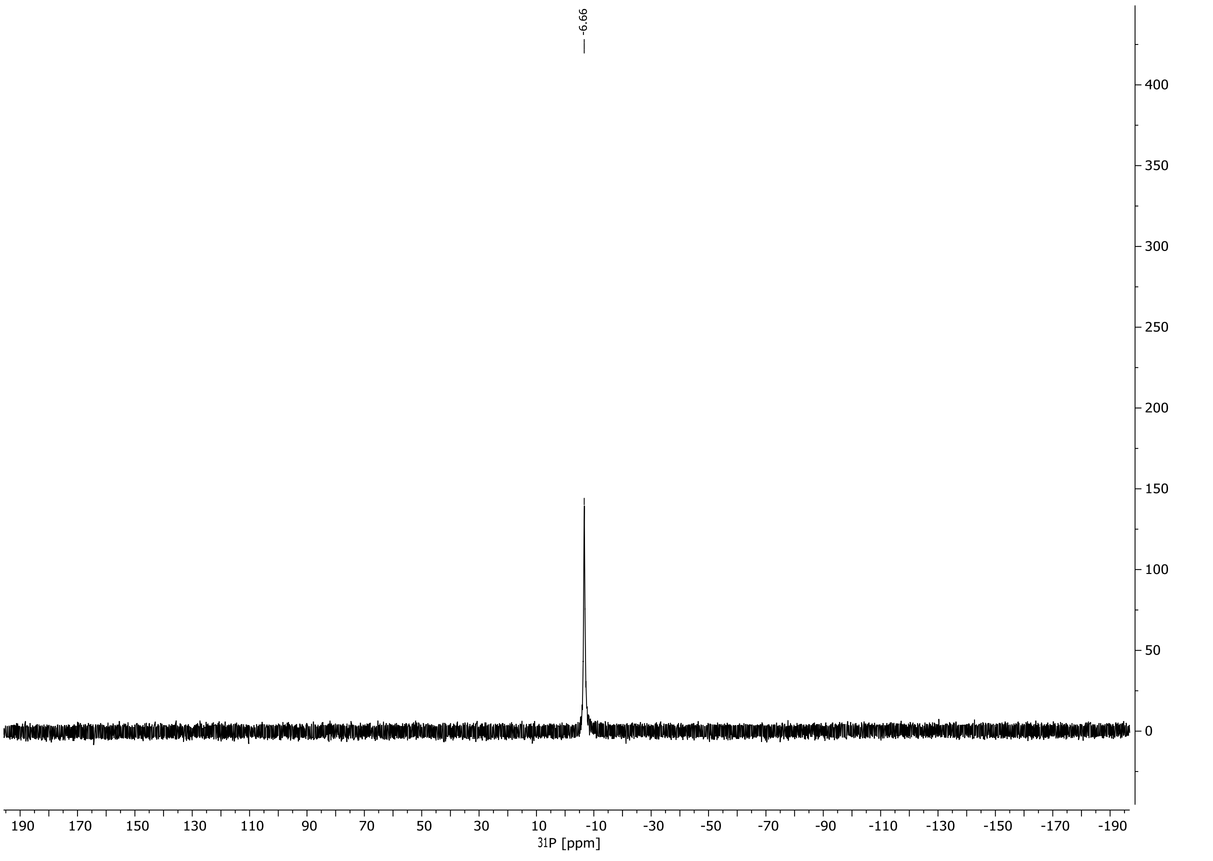

^{13}C NMR of compound **8a**

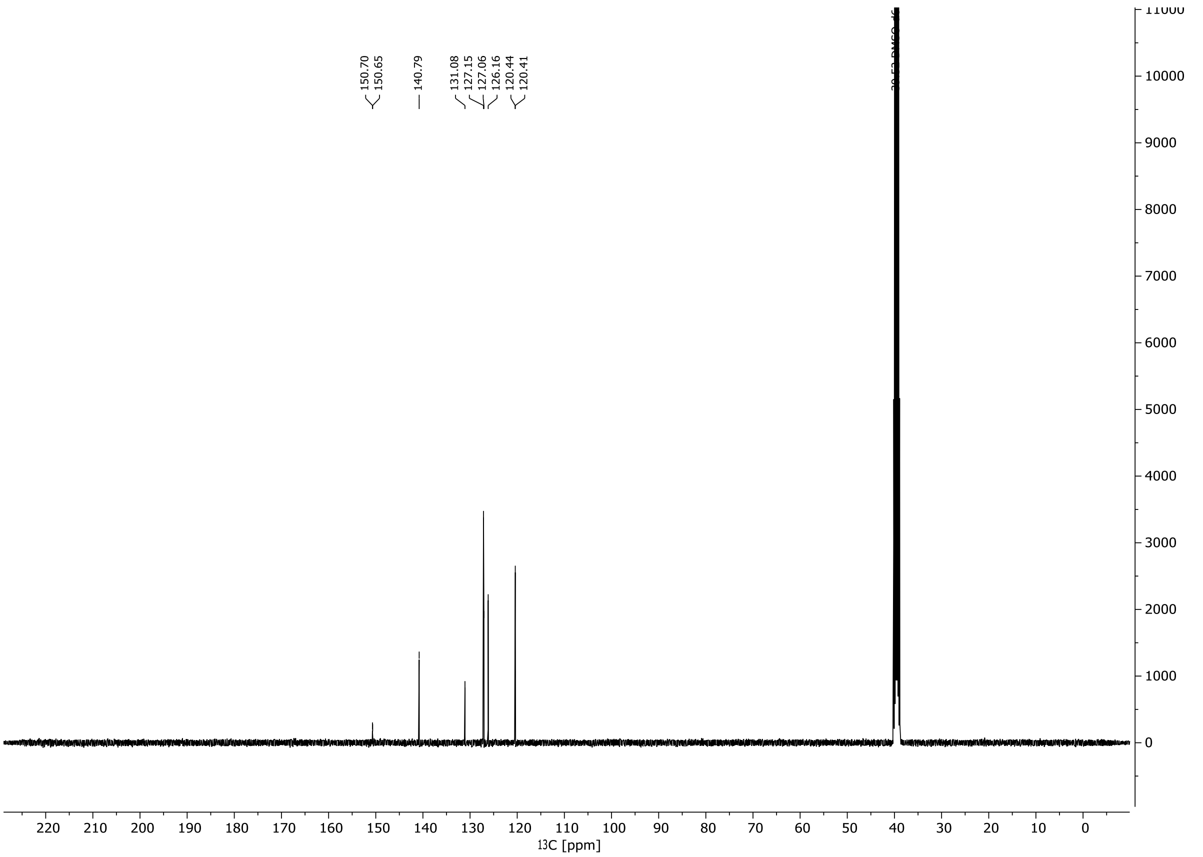

^{1}H NMR of compound **8b**

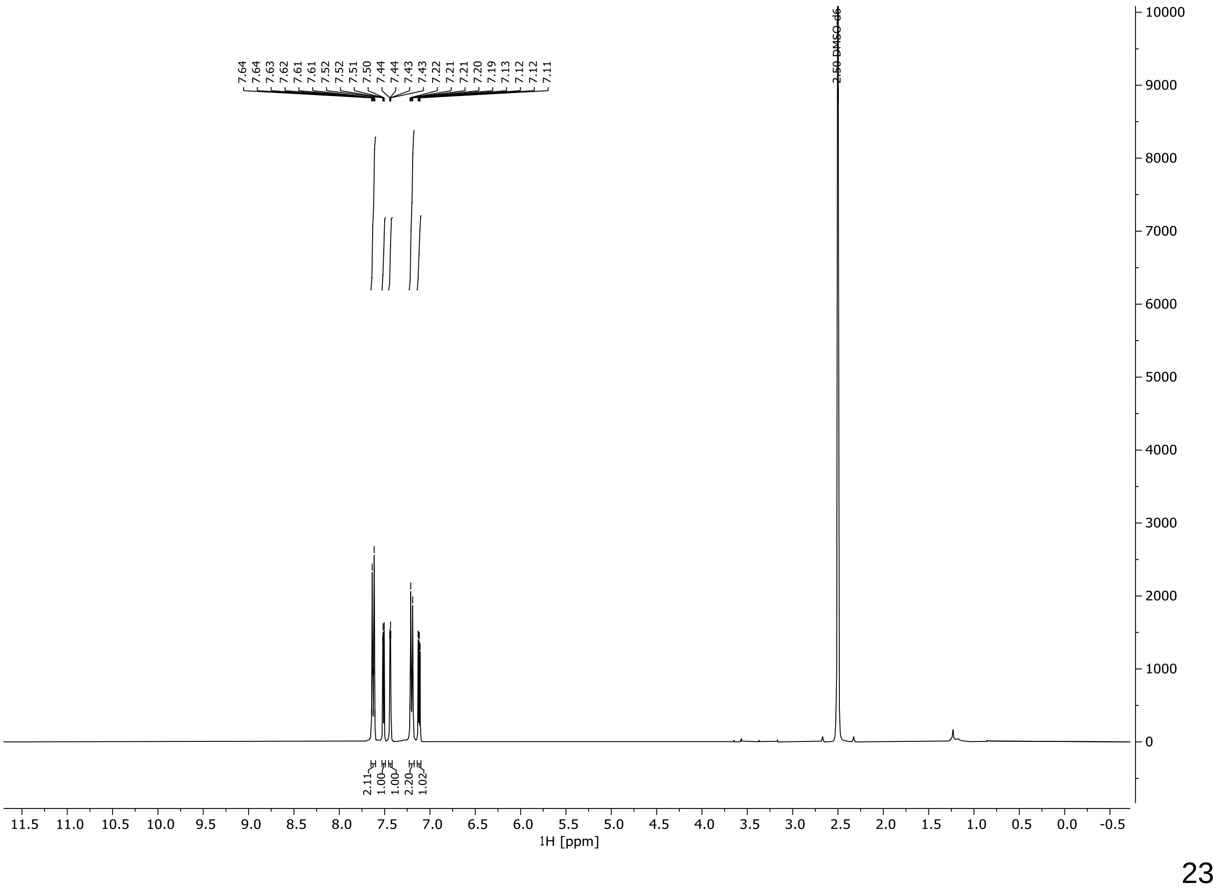

^{31}P NMR of compound **8b**

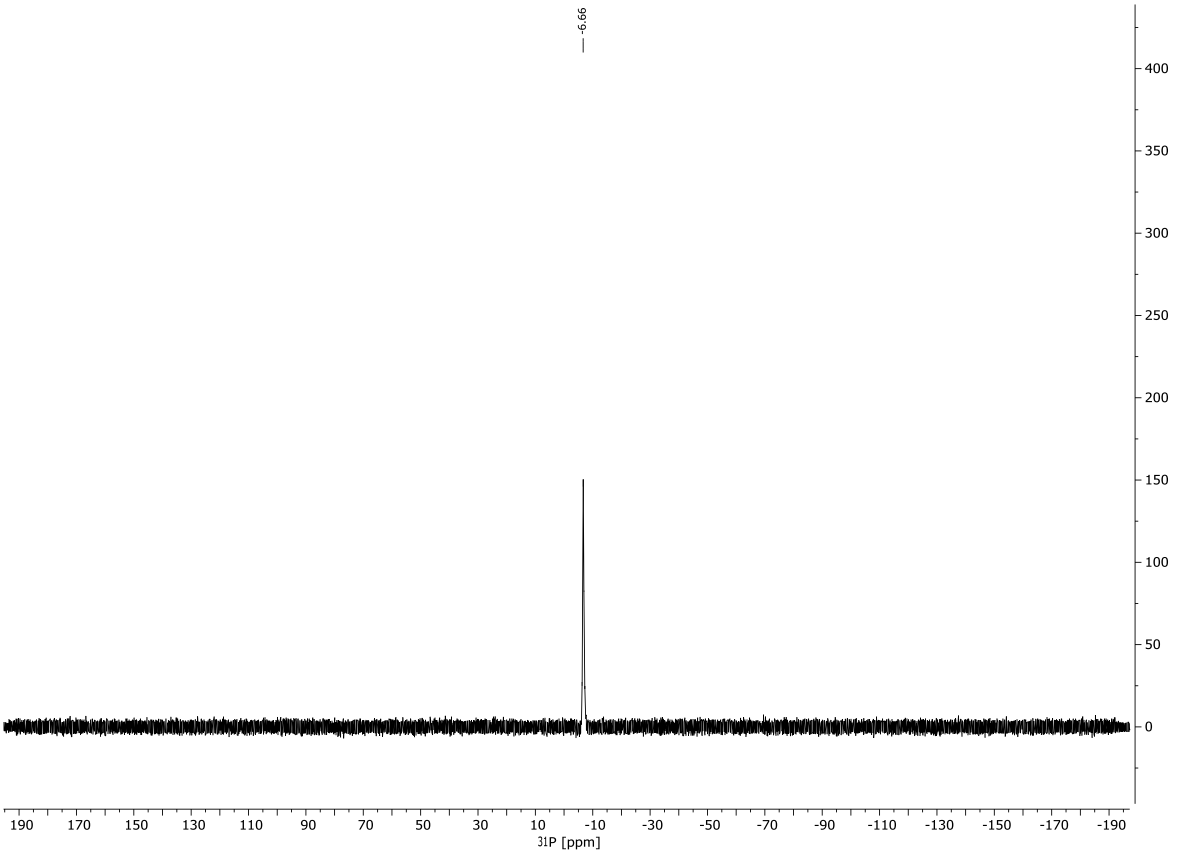

^{13}C NMR of compound **8b**

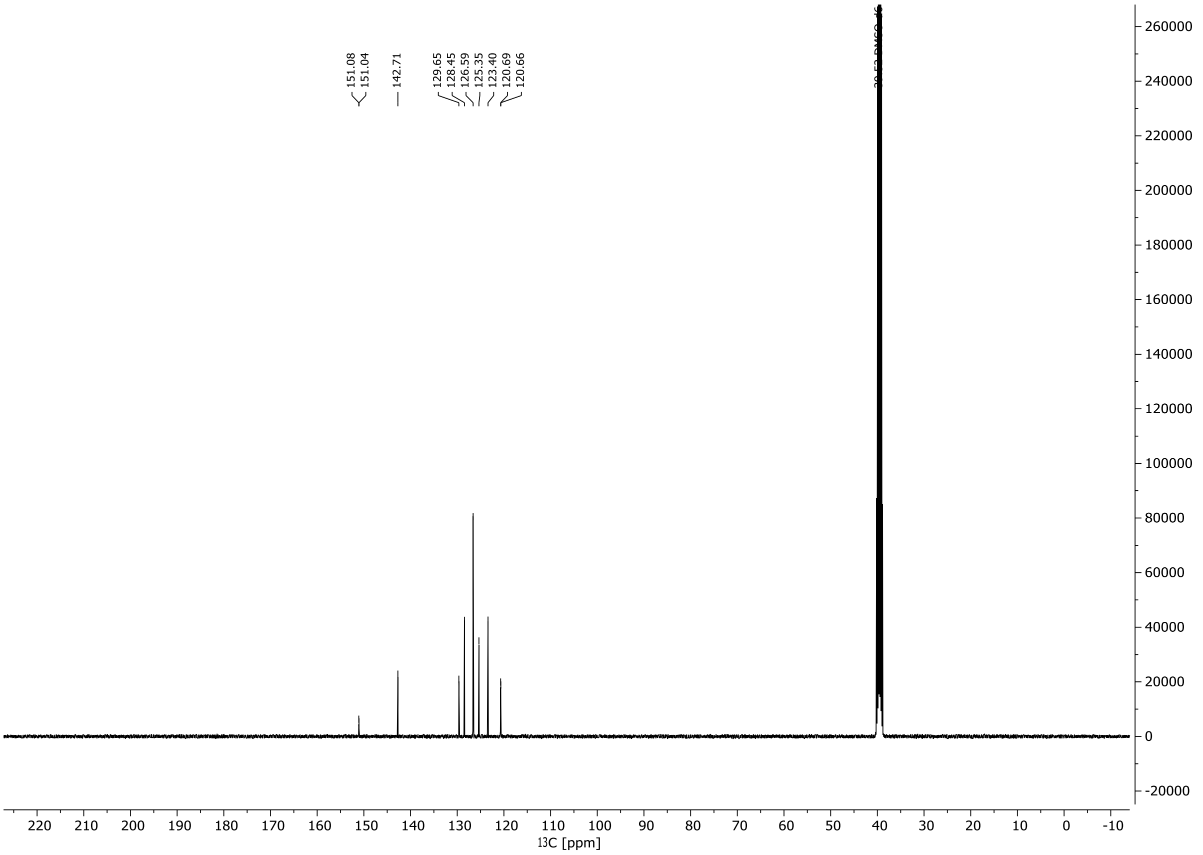

^{1}H NMR of compound **8c**

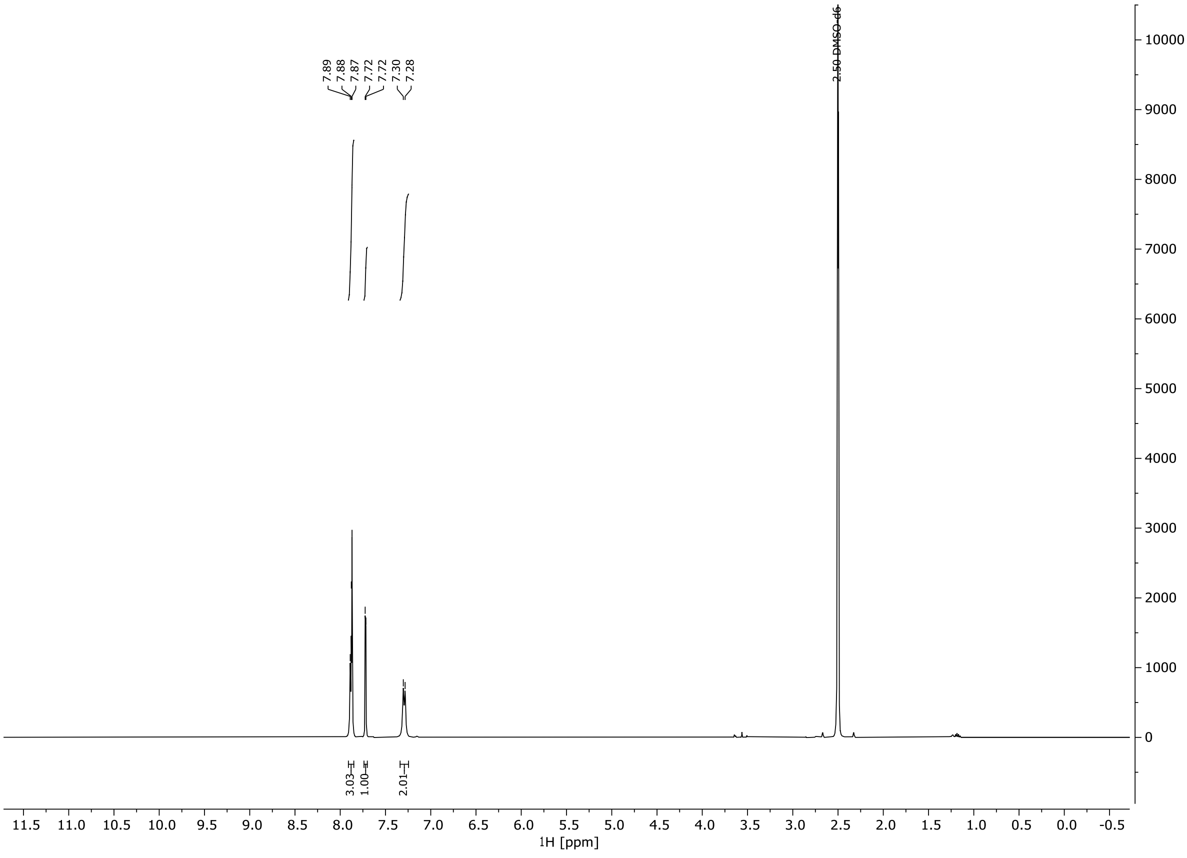

^{31}P NMR of compound **8c**

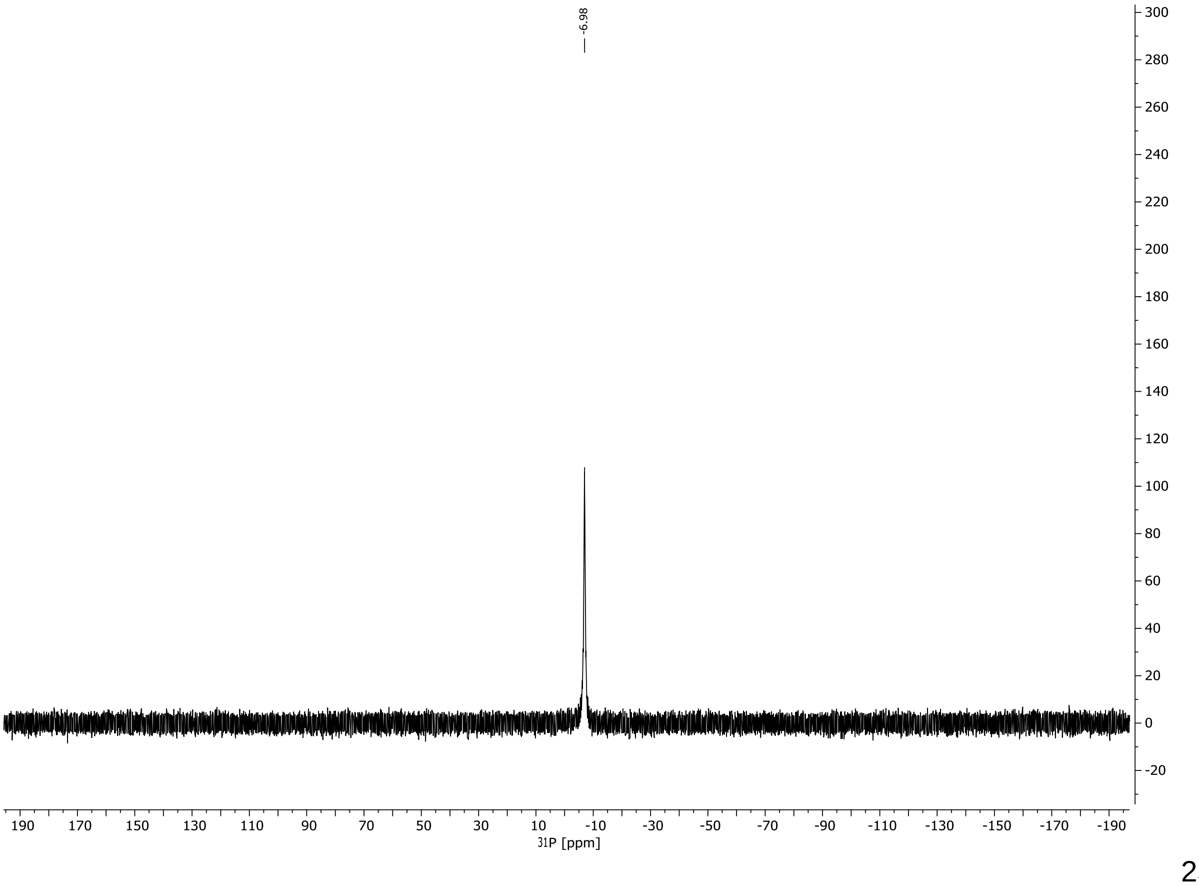

^{13}C NMR of compound **8c**

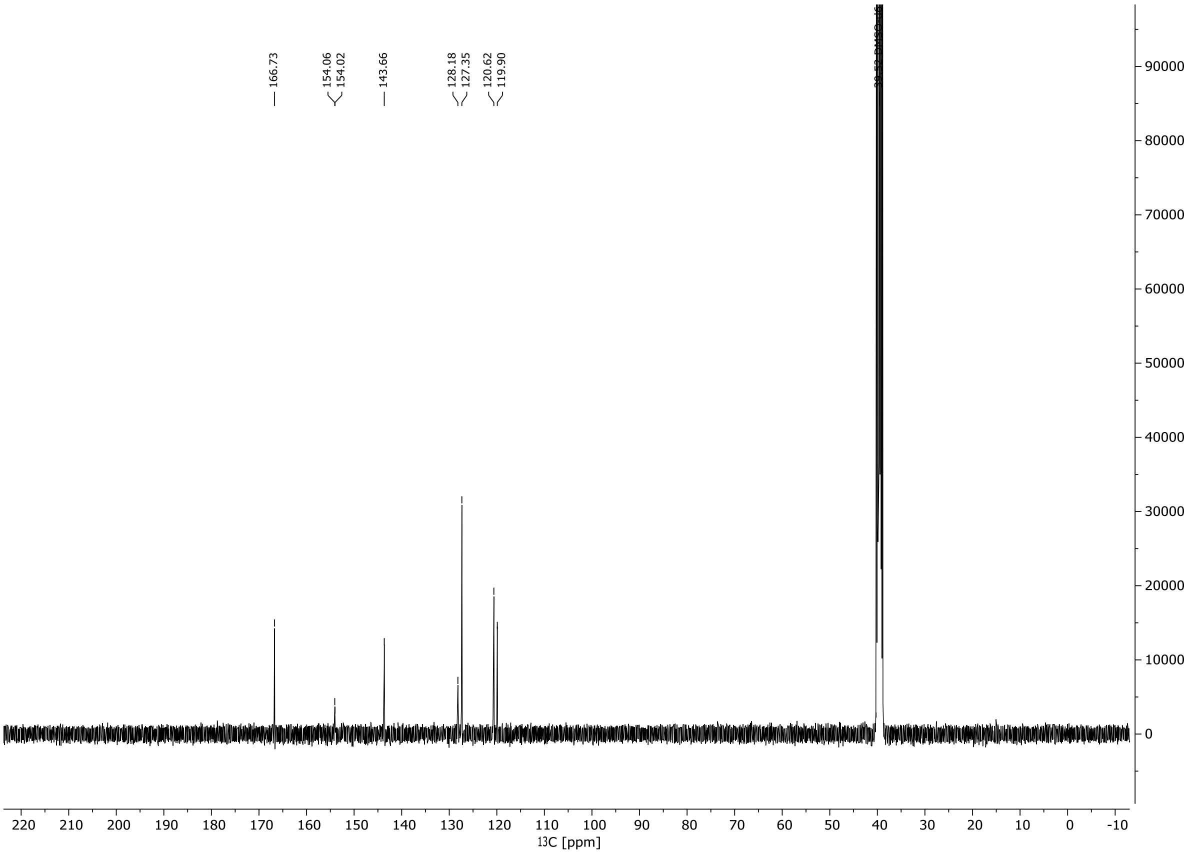

^{1}H NMR of compound **11**

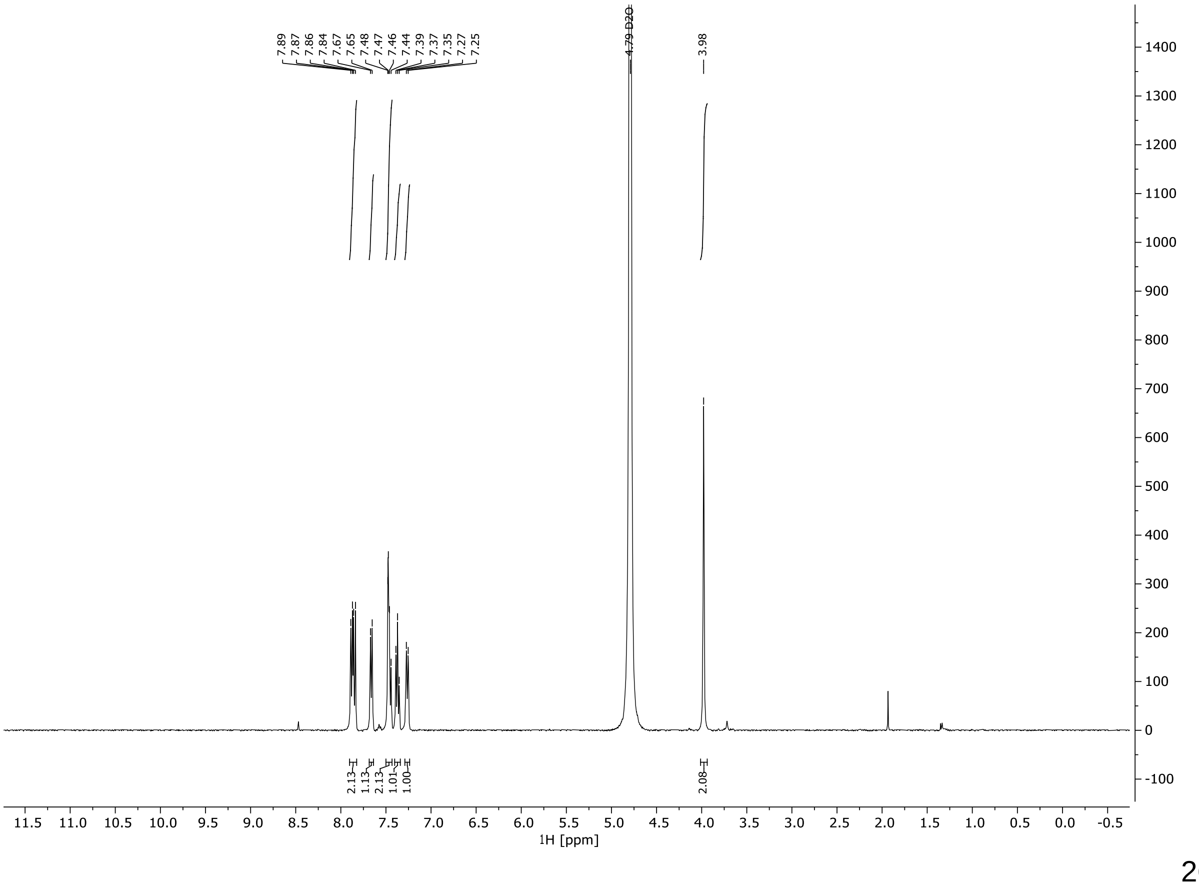

^{31}P NMR of compound **11**

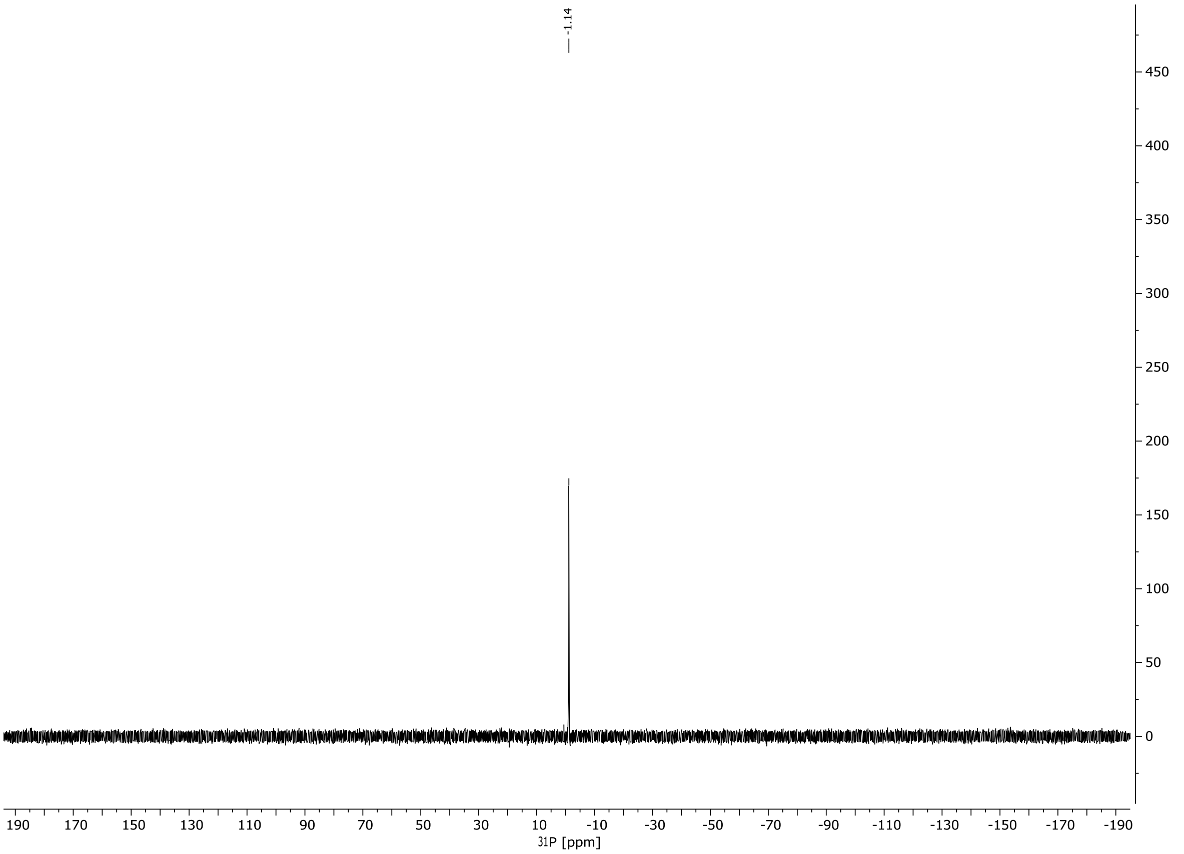

^{13}C NMR of compound **11**

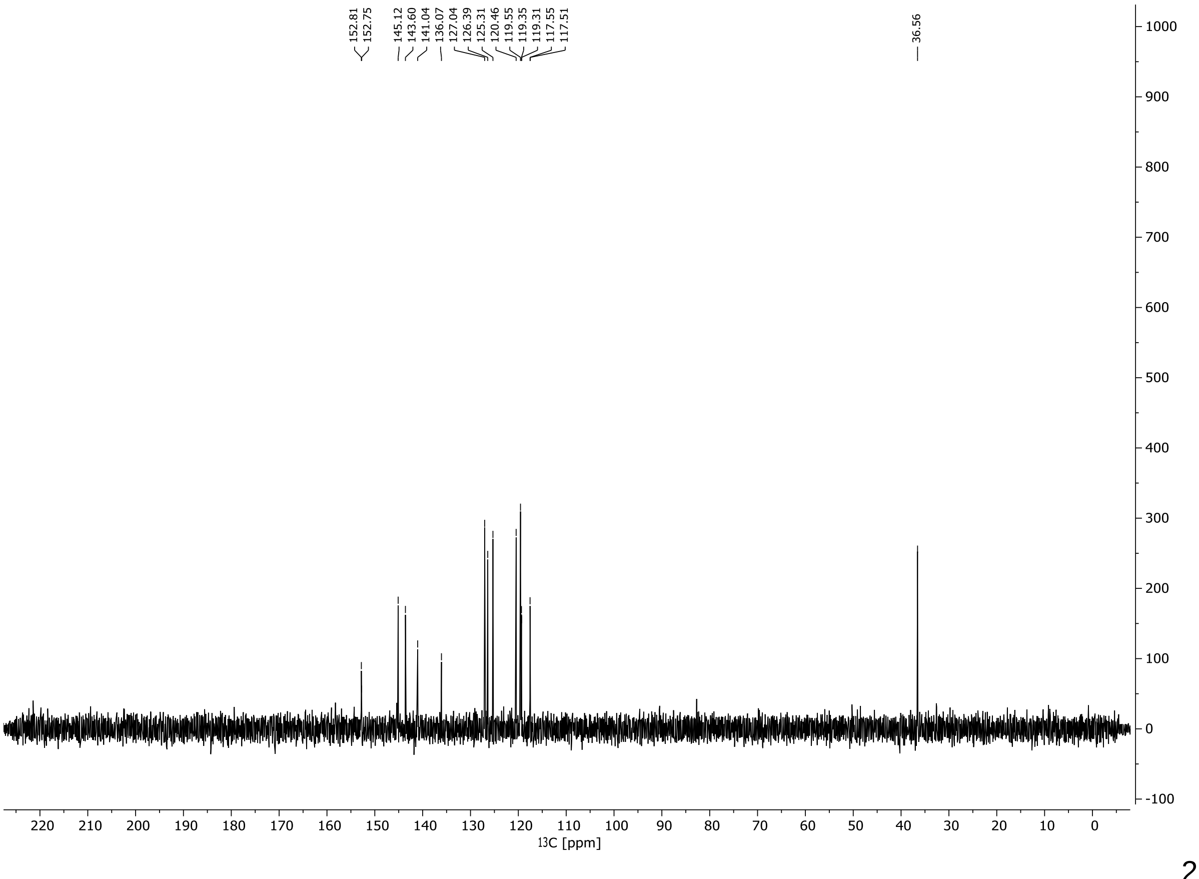

^{1}H NMR of compound **12**

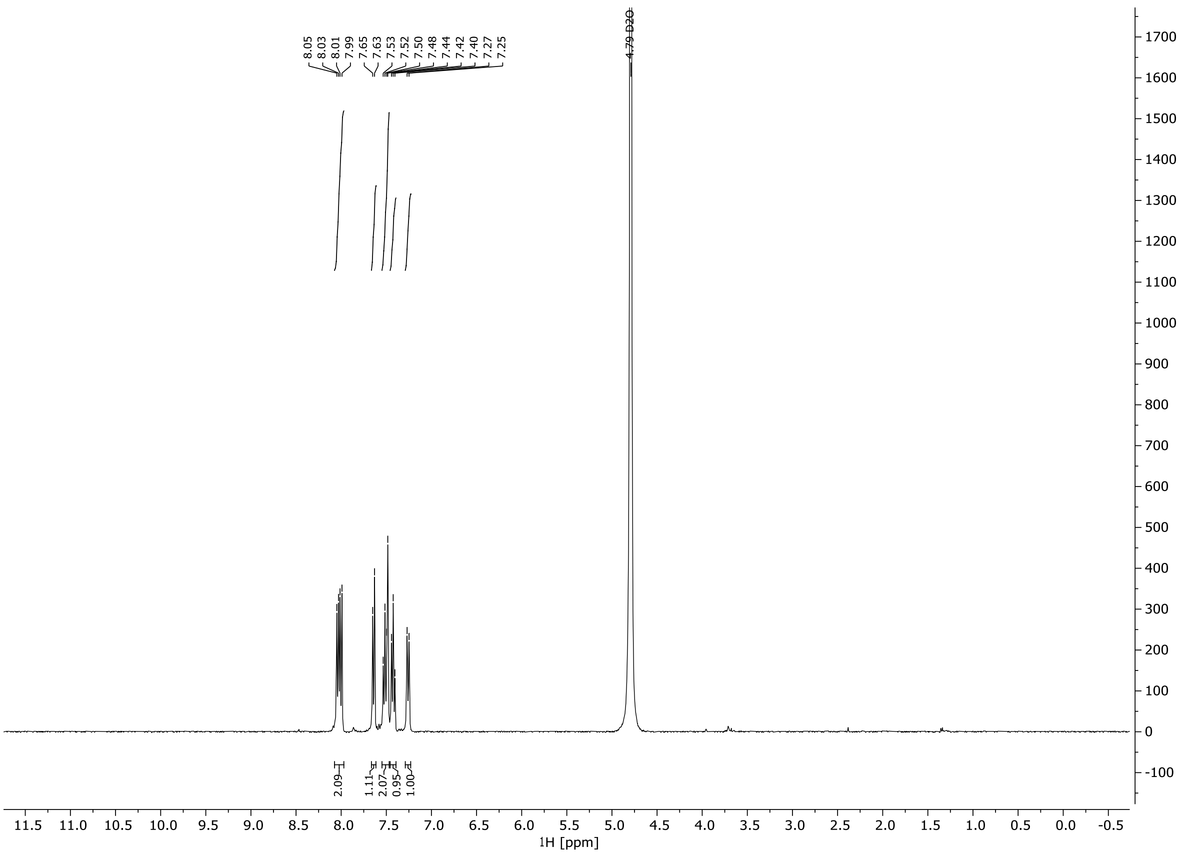

^{31}P NMR of compound **12**

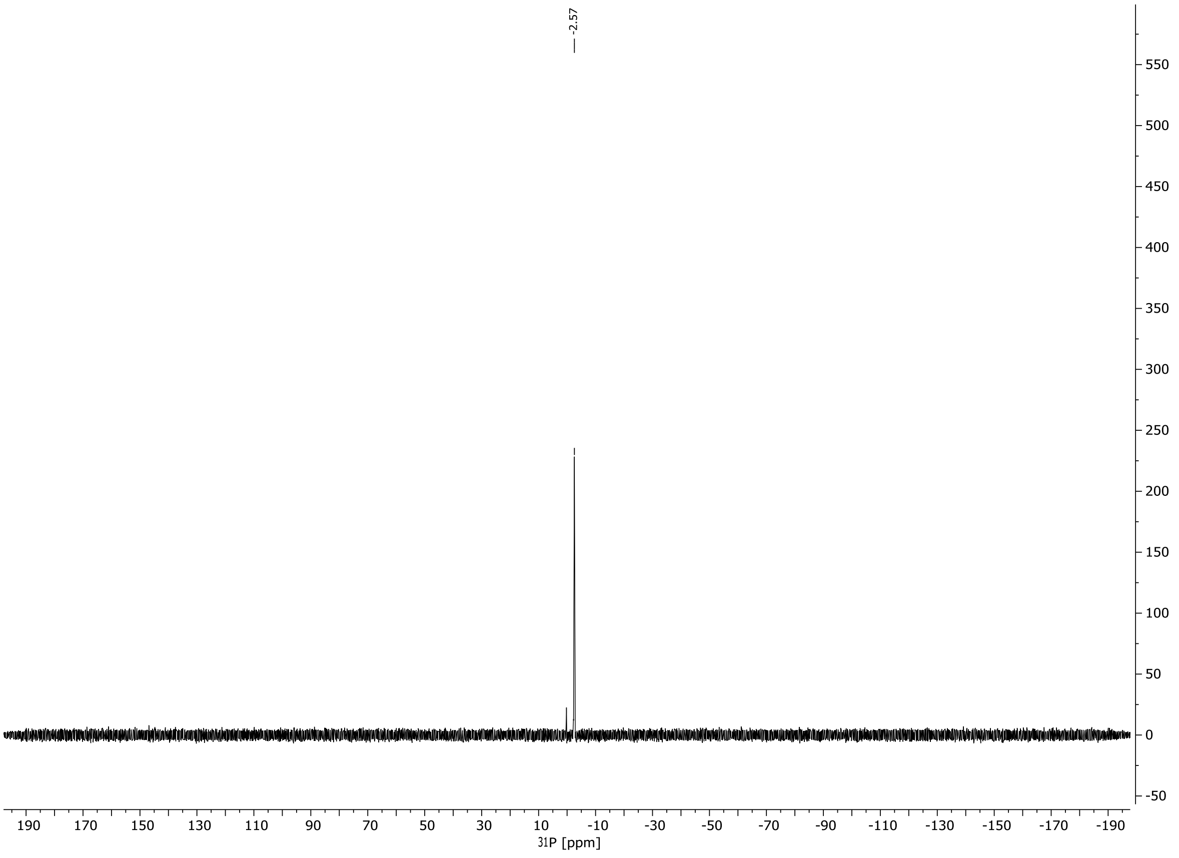

^{13}C NMR of compound **12**

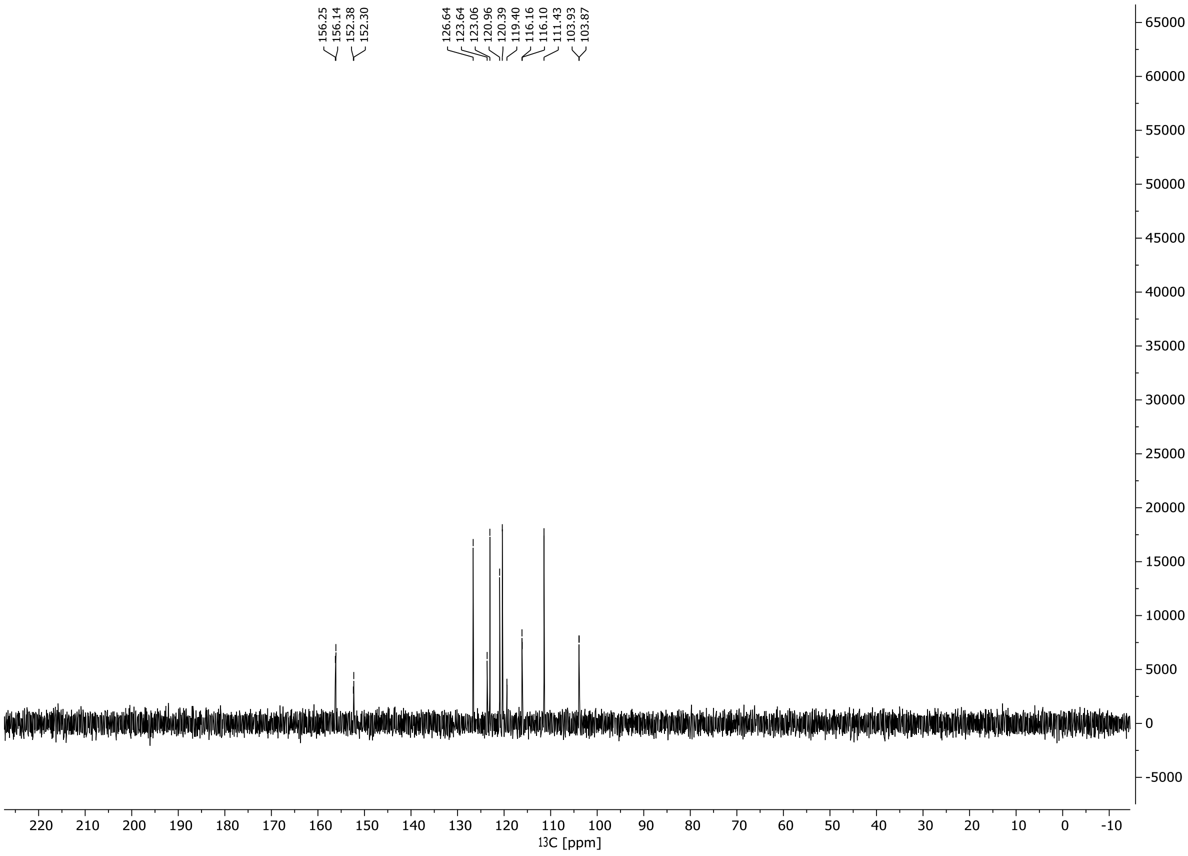

^{1}H NMR of compound **8d**

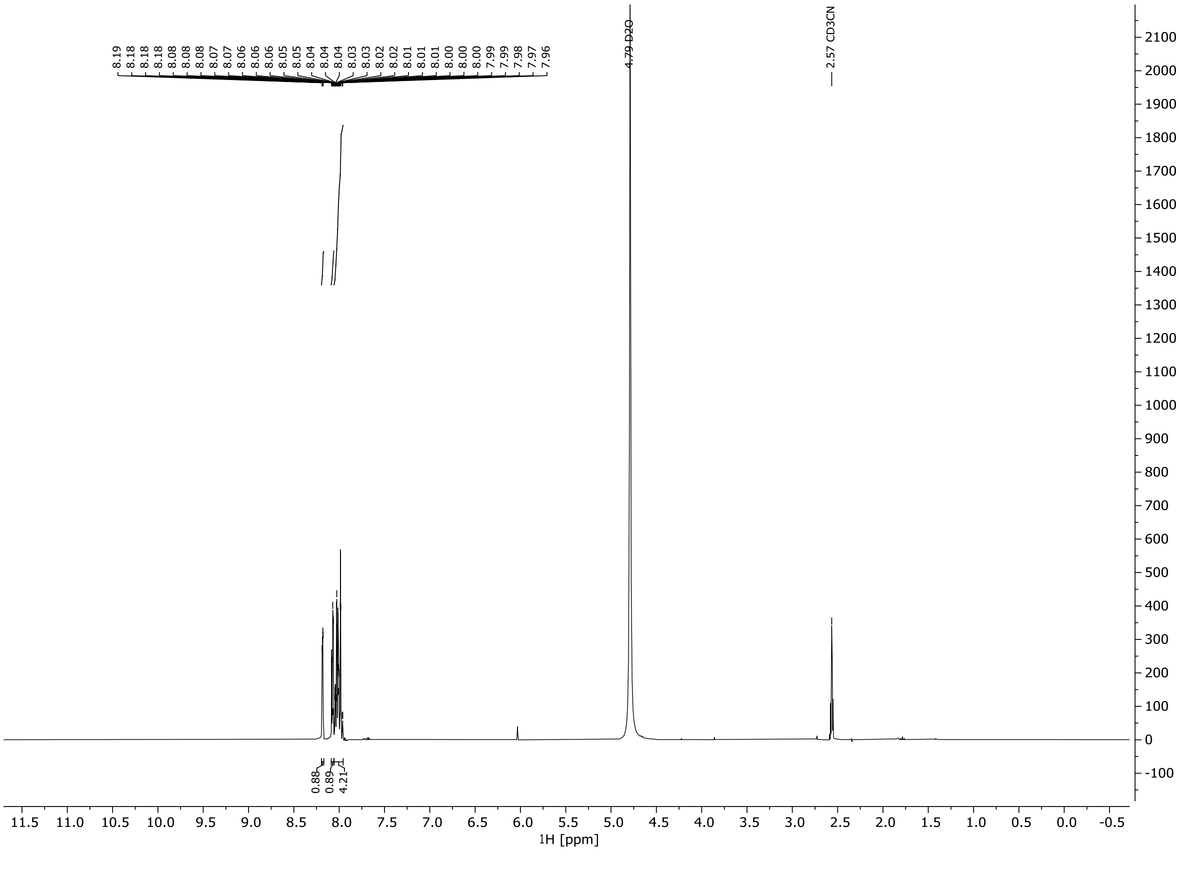

^{19}F NMR of compound **8d**

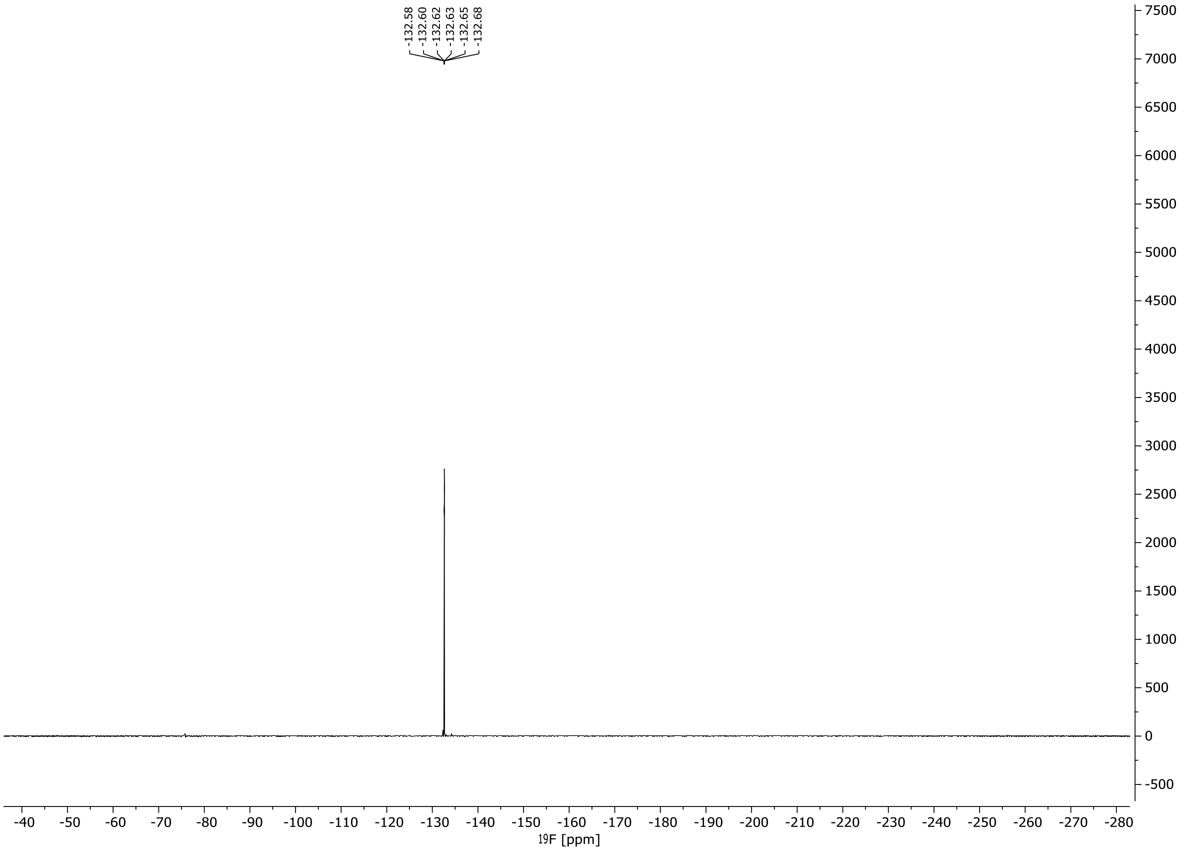

^{31}P NMR of compound **8d**

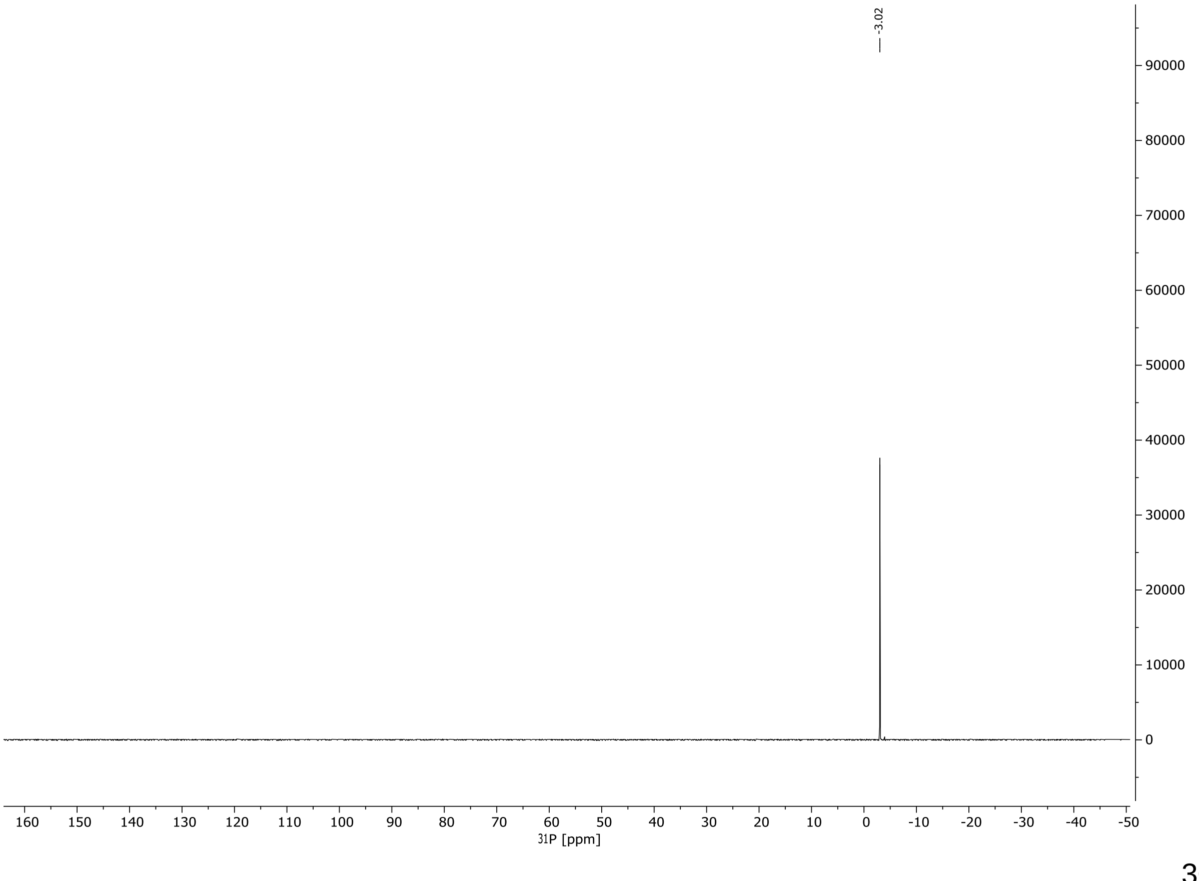

^{13}C NMR of compound **8d**

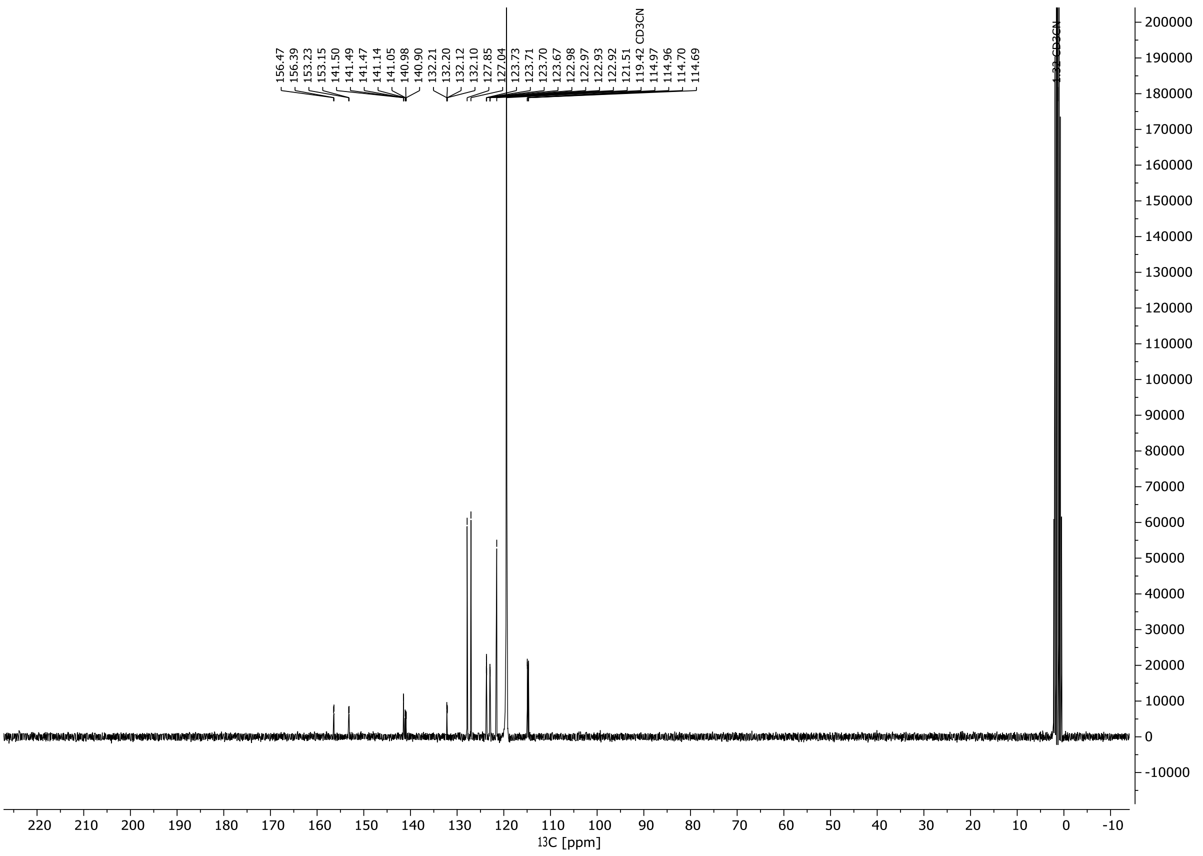

^{1}H NMR of compound **8e**

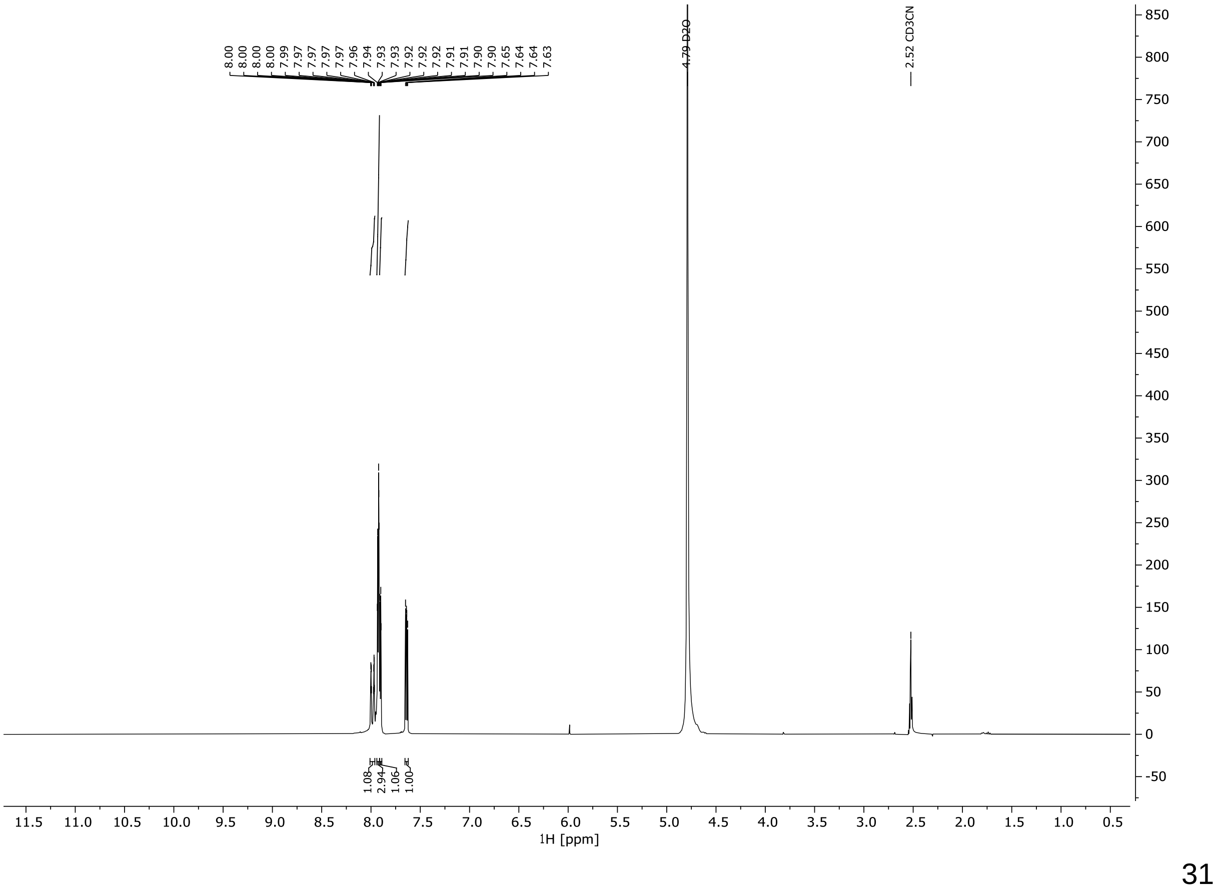

^{19}F NMR of compound **8e**

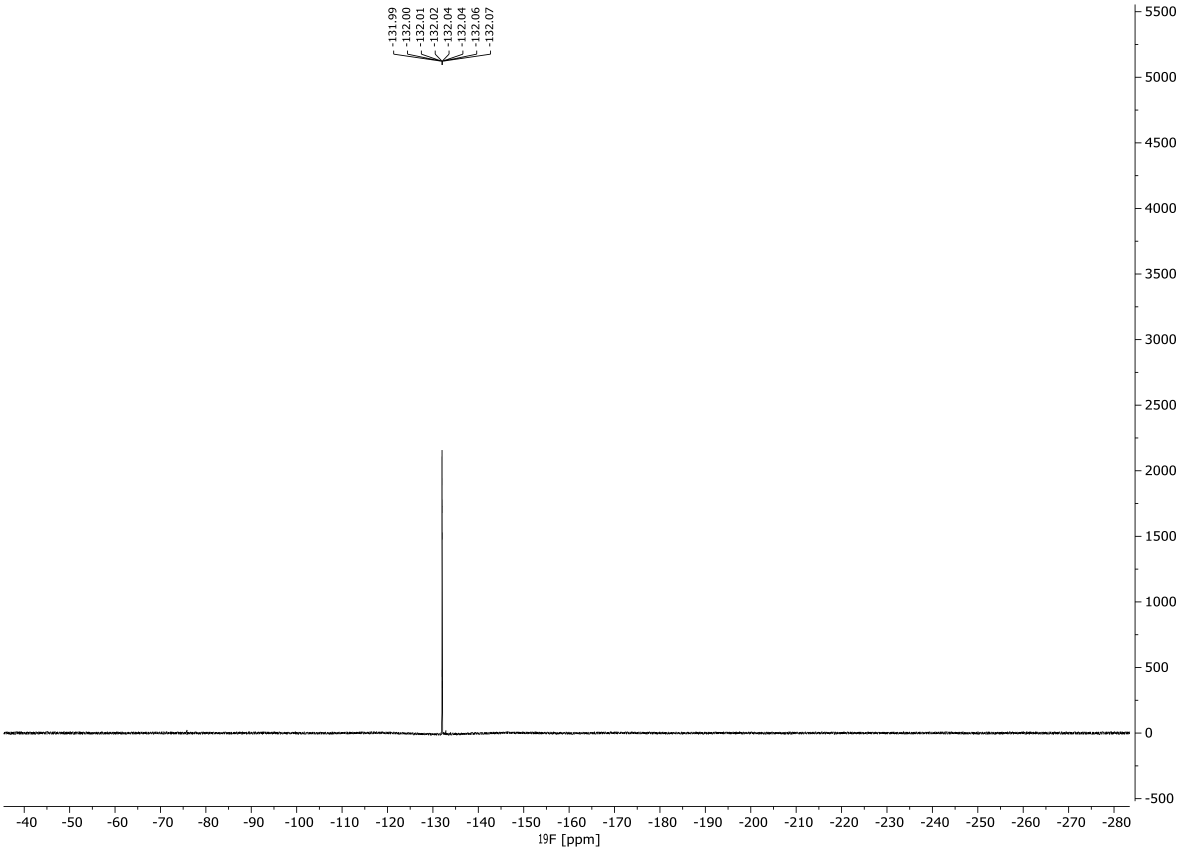

^{31}P NMR of compound **8e**

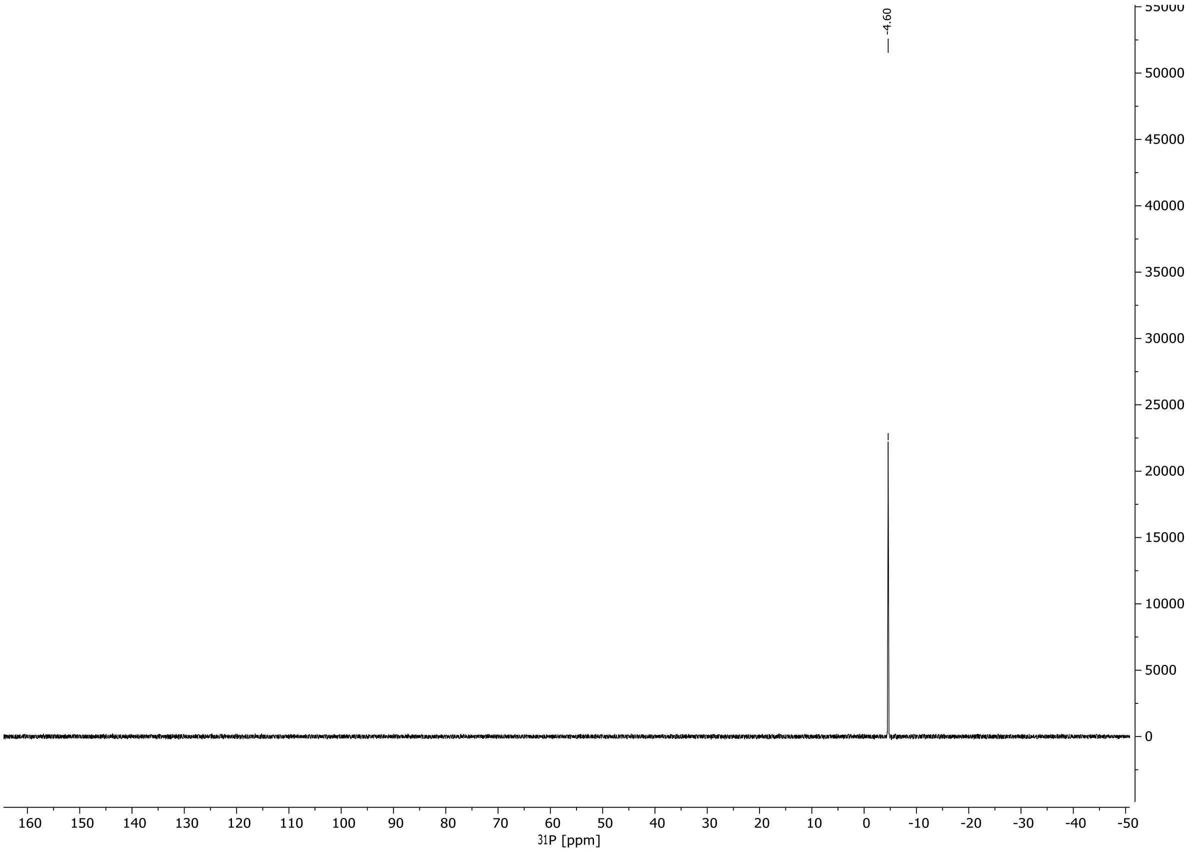

^{13}C NMR of compound **8e**

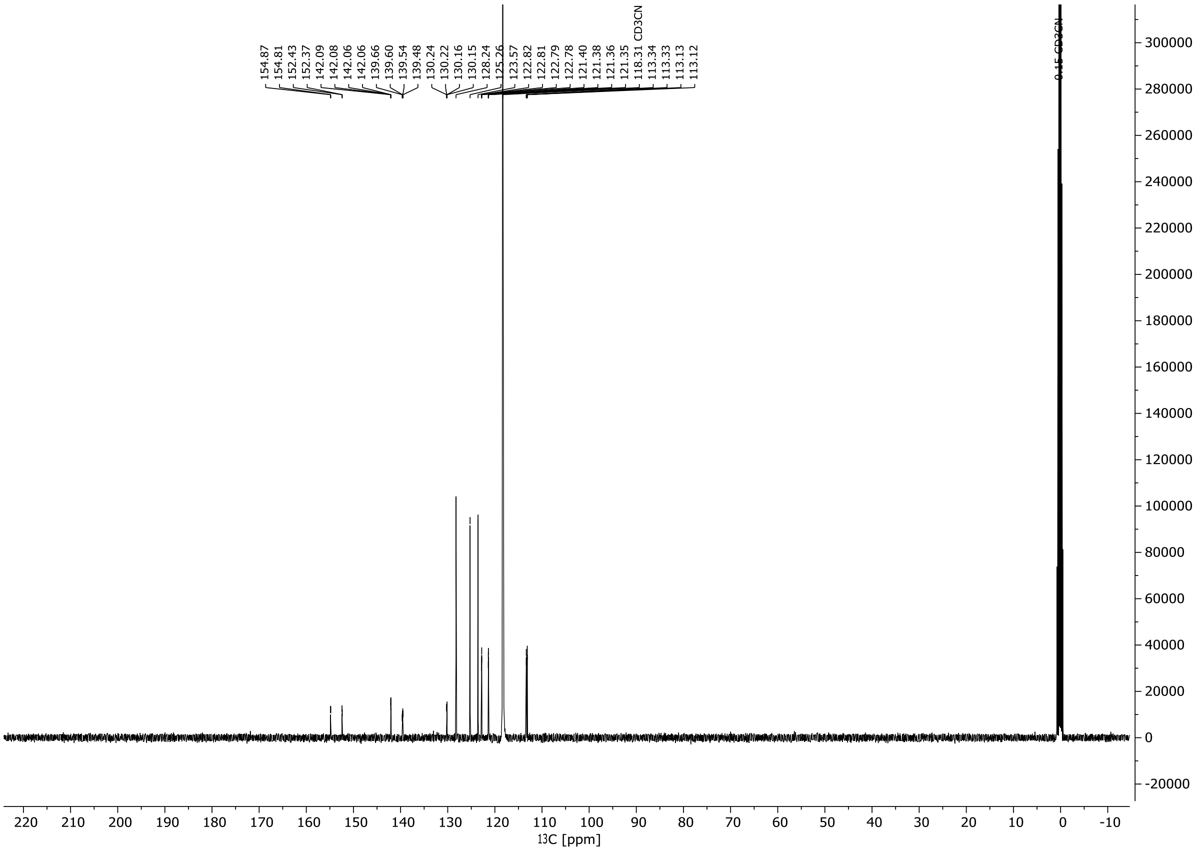

^{1}H NMR of compound **8f**

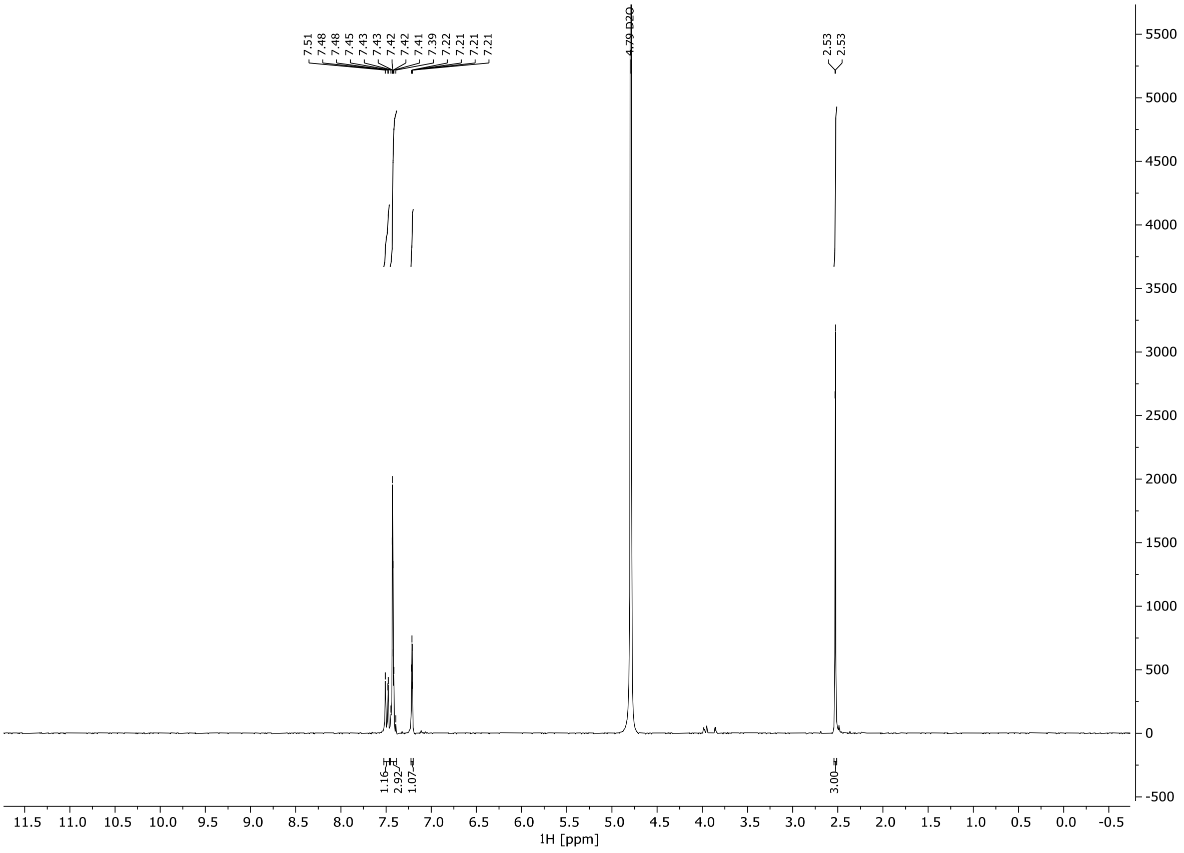

^{19}F NMR of compound **8f**

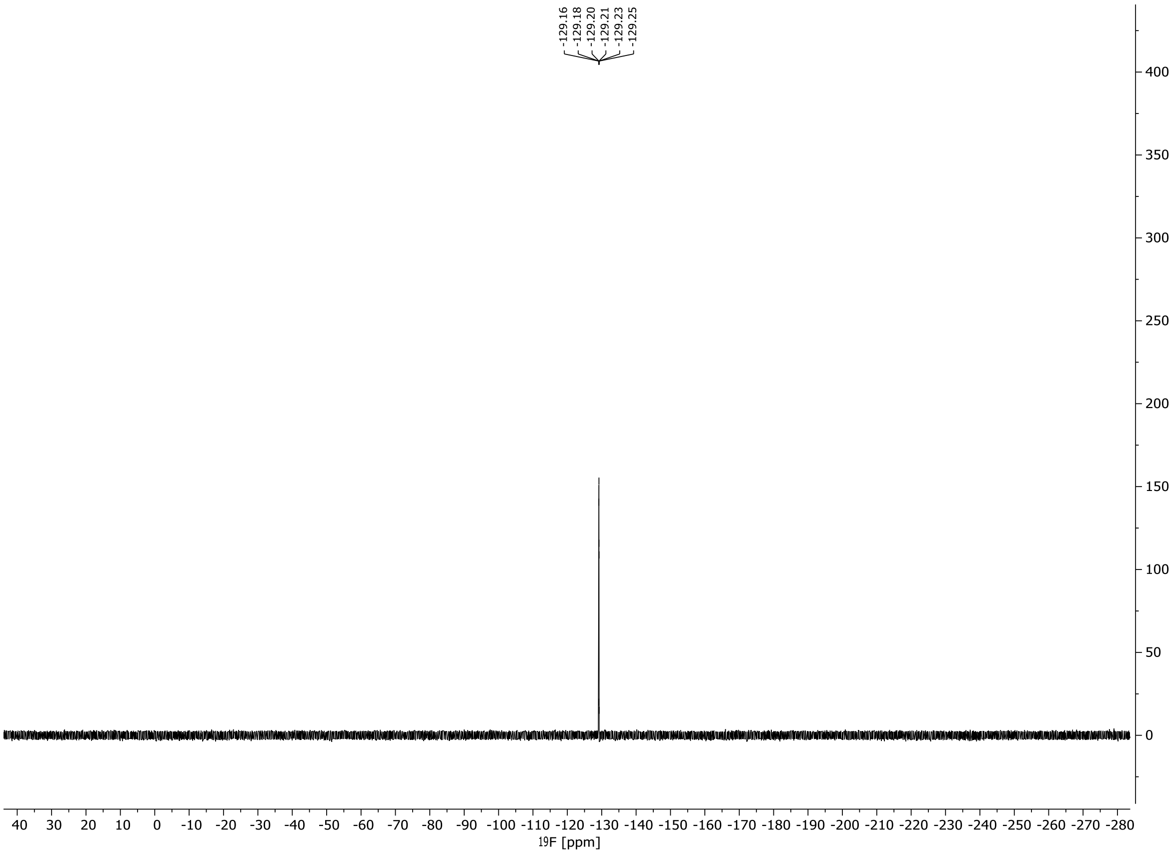

^{31}P NMR of compound **8f**

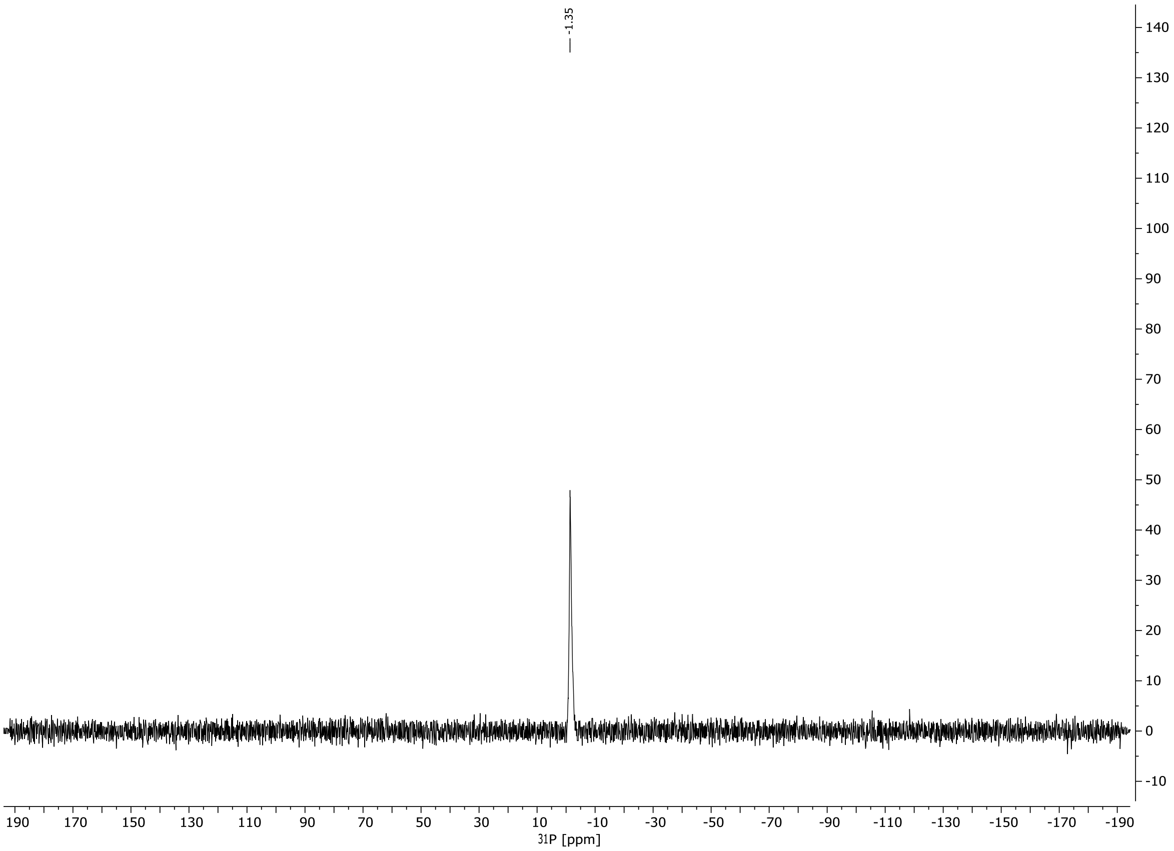

^{13}C NMR of compound **8f**

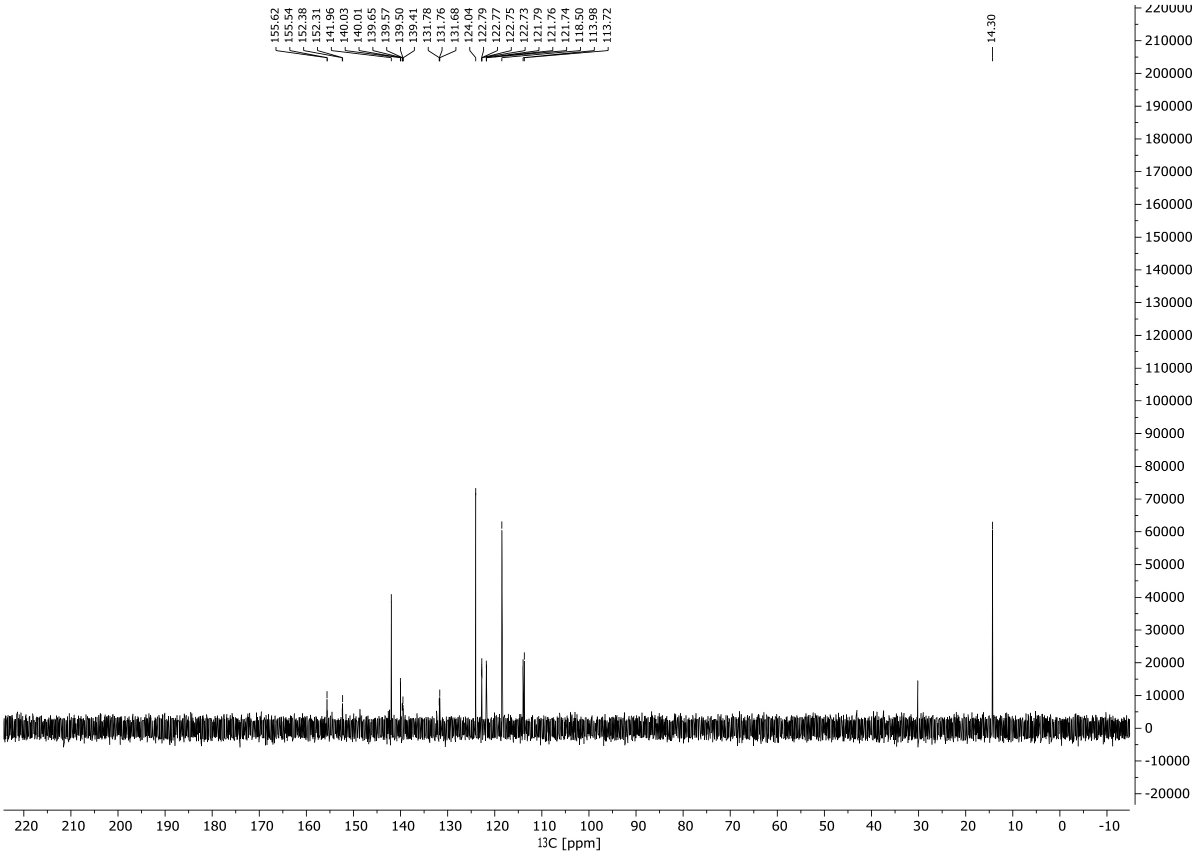

Supporting references

[1] N. Brovchenko, A. Berg, S. Schubert, J. Grab, T. Munzel, C. Protzel, K. Natarajan, T. Berg "Biaryl Phosphates and Phosphonates as Selective Inhibitors of the Transcription Factor STAT4", *Angew. Chem. Int. Ed.* **2025**, *64*, e202504420.

[2] Z. Nikolovska-Coleska, R. Wang, X. Fang, H. Pan, Y. Tomita, P. Li, P. P. Roller, K. Krajewski, N. G. Saito, J. A. Stuckey, S. Wang "Development and optimization of a binding assay for the XIAP BIR3 domain using fluorescence polarization", *Anal. Biochem.* **2004**, *332*, 261-273.

[3] S. Keller, C. Vargas, H. Zhao, G. Piszczek, C. A. Brautigam, P. Schuck "High-precision isothermal titration calorimetry with automated peak-shape analysis", *Anal. Chem.* **2012**, *84*, 5066-5073.

[4] T. H. Scheuermann, C. A. Brautigam "High-precision, automated integration of multiple isothermal titration calorimetric thermograms: new features of NITPIC", *Methods* **2015**, *76*, 87-98.

[5] J. C. D. Houtman, P. H. Brown, B. Bowden, H. Yamaguchi, E. Appella, L. E. Samelson, P. Schuck "Studying multisite binary and ternary protein interactions by global analysis of isothermal titration calorimetry data in SEDPHAT: Application to adaptor protein complexes in cell signaling", *Protein Sci.* **2007**, *16*, 30-42.

[6] C. A. Brautigam, in *Methods Enzymol., Vol. 562* (Ed.: J. L. Cole), Academic Press, **2015**, pp. 109-133.
